# Conserved structures and dynamics in 5′-proximal regions of Betacoronavirus RNA genomes

**DOI:** 10.1093/nar/gkae144

**Published:** 2024-03-01

**Authors:** Tales Rocha de Moura, Elżbieta Purta, Agata Bernat, Eva M Martín-Cuevas, Małgorzata Kurkowska, Eugene F Baulin, Sunandan Mukherjee, Jakub Nowak, Artur P Biela, Michał Rawski, Sebastian Glatt, Fernando Moreno-Herrero, Janusz M Bujnicki

**Affiliations:** Laboratory of Bioinformatics and Protein Engineering, International Institute of Molecular and Cell Biology in Warsaw, ul. Ks. Trojdena 4, 02-109 Warsaw, Poland; Laboratory of Bioinformatics and Protein Engineering, International Institute of Molecular and Cell Biology in Warsaw, ul. Ks. Trojdena 4, 02-109 Warsaw, Poland; Laboratory of Bioinformatics and Protein Engineering, International Institute of Molecular and Cell Biology in Warsaw, ul. Ks. Trojdena 4, 02-109 Warsaw, Poland; Department of Macromolecular Structures, Centro Nacional de Biotecnología, Consejo Superior de Investigaciones Científicas, Madrid, Spain; Laboratory of Bioinformatics and Protein Engineering, International Institute of Molecular and Cell Biology in Warsaw, ul. Ks. Trojdena 4, 02-109 Warsaw, Poland; Laboratory of Bioinformatics and Protein Engineering, International Institute of Molecular and Cell Biology in Warsaw, ul. Ks. Trojdena 4, 02-109 Warsaw, Poland; Laboratory of Bioinformatics and Protein Engineering, International Institute of Molecular and Cell Biology in Warsaw, ul. Ks. Trojdena 4, 02-109 Warsaw, Poland; Malopolska Centre of Biotechnology, Jagiellonian University, Krakow, Poland; Malopolska Centre of Biotechnology, Jagiellonian University, Krakow, Poland; Malopolska Centre of Biotechnology, Jagiellonian University, Krakow, Poland; National Synchrotron Radiation Centre SOLARIS, Jagiellonian University, Krakow, Poland; Malopolska Centre of Biotechnology, Jagiellonian University, Krakow, Poland; Department of Macromolecular Structures, Centro Nacional de Biotecnología, Consejo Superior de Investigaciones Científicas, Madrid, Spain; Laboratory of Bioinformatics and Protein Engineering, International Institute of Molecular and Cell Biology in Warsaw, ul. Ks. Trojdena 4, 02-109 Warsaw, Poland

## Abstract

Betacoronaviruses are a genus within the Coronaviridae family of RNA viruses. They are capable of infecting vertebrates and causing epidemics as well as global pandemics in humans. Mitigating the threat posed by Betacoronaviruses requires an understanding of their molecular diversity. The development of novel antivirals hinges on understanding the key regulatory elements within the viral RNA genomes, in particular the 5′-proximal region, which is pivotal for viral protein synthesis. Using a combination of cryo-electron microscopy, atomic force microscopy, chemical probing, and computational modeling, we determined the structures of 5′-proximal regions in RNA genomes of Betacoronaviruses from four subgenera: OC43-CoV, SARS-CoV-2, MERS-CoV, and Rousettus bat-CoV. We obtained cryo-electron microscopy maps and determined atomic-resolution models for the stem-loop-5 (SL5) region at the translation start site and found that despite low sequence similarity and variable length of the helical elements it exhibits a remarkable structural conservation. Atomic force microscopy imaging revealed a common domain organization and a dynamic arrangement of structural elements connected with flexible linkers across all four Betacoronavirus subgenera. Together, these results reveal common features of a critical regulatory region shared between different Betacoronavirus RNA genomes, which may allow targeting of these RNAs by broad-spectrum antiviral therapeutics.

## Graphical Abstract

**Graphical Abstract:**
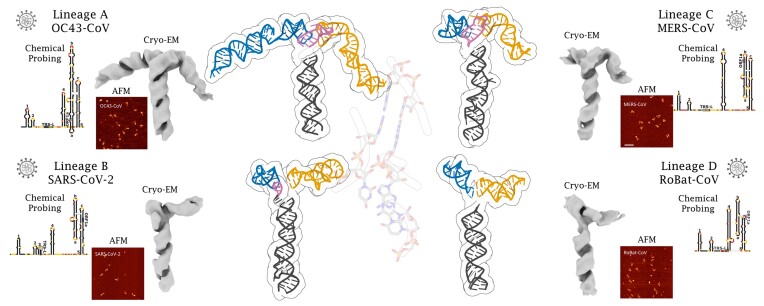

## Introduction

Coronaviruses are large, enveloped, positive-stranded RNA viruses. Among them, Betacoronaviruses (βCoVs) have attracted worldwide attention due to their pathogenic capacity and history of causing epidemics and global pandemics. The βCoV genus is divided into five evolutionarily distinct subgenera A (*Embecovirus*), B (*Sarbecovirus*), C (*Merbecovirus*), D (*Nobecovirus*), and E (*Hibecovirus*), of which the first four have multiple members and are well characterized (1). The highly pathogenic viruses that have recently caused severe acute respiratory diseases in humans belong to subgenus B (SARS-CoV and SARS-CoV-2), and C (MERS-CoV). Subgenus A includes viruses OC43 and HKU1 that currently cause the common cold in humans, while the ancestor of OC43-CoV is suspected of having caused the ‘Russian flu’ pandemic in 1889 (2). In subgenus D, viruses infecting humans have not yet been identified, however it includes bat-infecting viruses, e.g. Rousettus bat coronavirus HKU9 (RoBat-CoV). Bat Hp-betacoronavirus Zhejiang2013, found in the bat *Hipposideros pratti*, is the only member of the subgenus E and it remains poorly characterized (3). Bats are the natural reservoir for a large number and diversity of coronaviruses, including the ancestral viruses in subgenera B and C that gave rise to SARS-CoV and MERS-CoV (4). Only lineage A βCoVs have not been detected in bats.

Recurring outbreaks of βCoV infections in humans pose serious threats to global public health and can cause enormous socioeconomic disruptions. In particular, the emergence of the COVID-19 pandemic caused by SARS-CoV-2 has highlighted the urgent need to better understand the molecular mechanisms of βCoV action. Combating the βCoV threat to humans requires an understanding of the molecular diversity of all types of these viruses, including the presence of common functional elements that could become targets of broad-spectrum antiviral drugs. In particular, the identification of molecular structures conserved across βCoVs from different subgenera can guide the development of diagnostic and antiviral strategies targeting all these viruses, including new human-infecting βCoVs that will emerge in the future.

Coronavirus genomes with a size of 25–31 kb are among the largest of all known RNA viruses. They encode viral proteins and contain several functional RNA elements, especially in the 5′- and 3′-proximal regions. These RNA elements regulate viral replication, RNA synthesis, and viral packaging. Several studies showed that RNA sequences in the 5′- and 3′-proximal regions of the βCoV RNA genome are essential for the virus (5). The results of various computational and experimental studies concluded that while the 5′-terminal genome regions of βCoVs exhibit extreme dissimilarity at the primary sequence level, different βCoVs harbor four elements of RNA secondary structure designated SL1, SL2, SL4, and SL5. An additional element SL3, was found in RNAs of viruses from subgenus B. SL1, SL2, SL3, and SL4 are simple stem-loop structures, whereas SL5 is predicted to contain three additional hairpin substructures (5a, 5b, and 5c) that form a junction (6–8).

Mechanistic models of the regulatory elements in the 5′-proximal regions of βCoV RNA genomes and their function currently remain speculative due to the paucity of 3D structural information. The determination of 3D structures for these 5′-terminal elements is important to understand how these elements function and whether they can be exploited in future therapeutic interventions. In addition, the structural comparison between regulatory RNA elements from SARS-CoV-2 and βCoVs from other subgenera is necessary to gain insight into what is most conserved among these dangerous pathogens.

Here, we present detailed structural analyses of the 5′-proximal regions of the RNA genome in representatives of the A, B, C, and D subgenera of βCoV, namely OC43-CoV, SARS-CoV-2, MERS-CoV, and RoBat-CoV, respectively. The results provide insights into the similarities and differences of the RNA secondary structure. We further determined the 3D structures of the SL5 elements from these representatives using single-particle cryo-EM, which revealed remarkable structural similarity despite the low sequence conservation and variable lengths. To further compare βCoVs, we examined the structure and dynamics of the 5′-proximal region using AFM and identified the SL5 element as essential for the overall structure of the 5′ region. We also conducted differential scanning fluorimetry and found similar thermal stabilities for the 5′-proximal regions and their corresponding SL5 elements in all representatives. Finally, through chemical probing at various temperatures coupled with computational analyses, we were able to identify the regions more susceptible to losing base pairing patterns and undergoing structural rearrangements in the SL5 of SARS-CoV-2.

## Materials and methods

### RNA sequence and alignment data

Genome sequences of βCoVs were obtained from the GenBank database and curated manually to select those with complete 5′ regions. Among these genomes, 13 belong to βCoV subgenus A, 9 to B, 11 to C, and 4 to D. Four individual alignments for each subgenus were constructed for the first 400 nucleotides of each sequence using LocaRNA (9), followed by manual curation to maximize the agreement with preliminary secondary structure predictions. Sequences with IDs: OK073091.1, MN908947.3, NC_019843.3, and NC_030886.1 were selected as representatives of subgenus A, B, C, and D, respectively. For the representative sequences, secondary structure prediction was performed using the RNAProbe web server, yielding a consensus from sequence-based structure prediction methods (10), then the secondary structure was adjusted based on the results of chemical probing, and ultimately, based on 3D structures determined by cryo-EM. The alignments of individual subgenera were then combined into a single structural alignment based on common patterns of secondary structure using LocaRNA (9), followed by manual curation. For the experimental analyses, representative sequences were truncated at their 3′ ends up to five nucleotides downstream of the SL5 element.

### RNA synthesis and purification

All RNAs used in this study were prepared by *in vitro* transcription. Templates for RNA synthesis (consisting of regions of interest followed by the 3′ end SHAPE cassette) were either purchased as dsDNA fragments (DNA strings, Thermo Fisher Scientific) or assembled in a PCR reaction using overlapping primers listed in Supplementary Materials (Supplementary Table S1). DNA templates were further amplified with primers specific for the region of interest (to obtain native RNA) or primers introducing the SHAPE cassette (for the synthesis of RNA used in chemical probing experiments). Regardless of the application, the T7 promoter sequence followed by two Gs was added at the 5′ end of the PCR products to ensure efficient transcription with T7 polymerase. Transcription was carried out using 50 ng/ul DNA in a buffer containing 1.5 mM NTPs mix, 40 mM Tris pH 8, 10 mM DTT, 2 mM spermidine 0.01% Triton-X100, 0.6 U/ul T7 polymerase (Thermo Scientific), and 1 U/ul RNase inhibitor (EURx) for 4 h at 37°C. The resulting RNA was purified by size exclusion chromatography (SEC) in MCB buffer (20 mM HEPES pH 7.5, 50 mM KCl, 50 mM NaCl, 1 mM MgCl, 1 mM DTT). Fractions containing homogeneous, monomeric RNA [as confirmed by native electrophoresis (11,12)] were pooled and concentrated on 10 kDa Amicon filters. The quality of RNA samples was checked by native and denaturing gel electrophoresis prior to further analysis.

### Chemical probing of RNA

**Figure 1.**
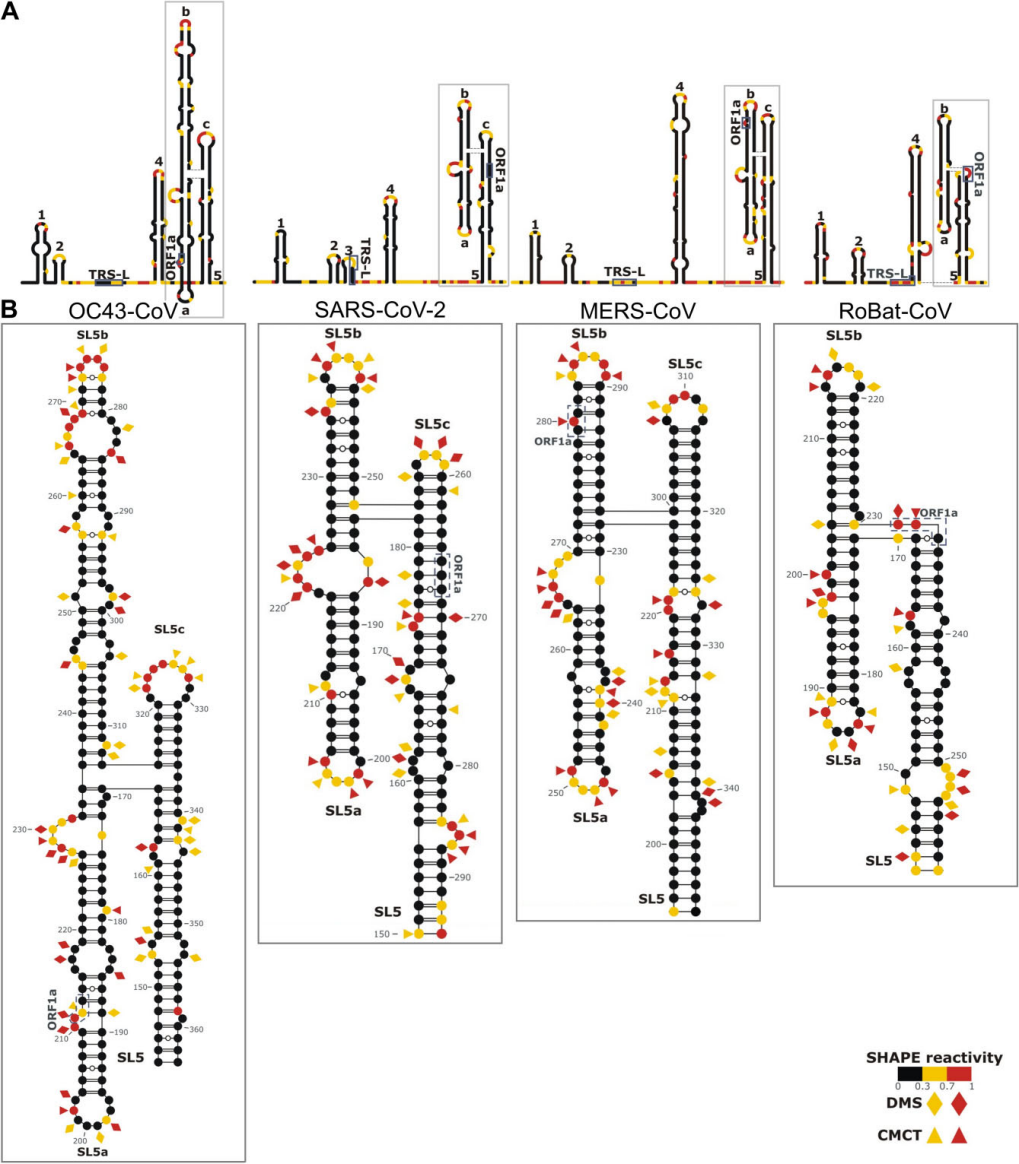
Secondary structure elements within the 5′-proximal regions of βCoVs from A, B, C, and D subgenera. (**A**) Schematic representations of the secondary structure of the full-length 5′-proximal regions. (**B**) Details of the secondary structure predictions for the SL5 element. Normalized reactivities across three biological replicates for the *in vitro* probing experiments are shown. Highly (red) and moderately (yellow) reactive residues from *in vitro* SHAPE (circles), DMS (diamonds), and CMCT (triangles) experiments are indicated. Highlighted features include stem-loops 1–5 (SL1–SL5), the leader transcriptional regulatory sequence (TRS-L) and the start codon of ORF1a. Substructures within SL5 are labeled as a, b, c. In SL5, the individual base pairs were manually edited to position them according to the 3D model (see below).

Chemical probing of RNA was performed as previously described (10,13), with minor modifications. The target RNA was incubated after refolding in the buffer containing 111 mM HEPES, pH 8.0, 6.6 mM MgCl_2_, 111 mM NaCl, with the probing reagent (positive reaction) or DMSO (control) under time and temperature conditions that allow a low level of modification with a given reagent: 2 pmol RNA was treated with either 1 ul of 65 mM NMIA (for 45 min at 37°C) or 25 mM DMS (for 5 min at room temperature) or 30 mM CMCT (for 15 min at room temperature). For temperature-dependent RNA modification experiments the RNA solution was equilibrated for 5 min at the reaction temperature (37–85°C), treated with NMIA and allowed to react for at least five hydrolysis half-lives (45–1.2 min for 37–85°C, respectively), calculated using the following formula: half-life(min) = 360 × exp[–0.102 × temperature (°C)] (13). Under the conditions used, when NMIA degradation proceeds to completion, no reactivity correction is required (14). Both modified and unmodified RNAs were reverse transcribed into cDNA using SuperScriptIII (Thermo Fisher Scientific) with VIC-labeled primers complementary to the 3′ end of the SHAPE cassette. In parallel, the dideoxy sequencing ladders were generated by primer extension on the unmodified RNA with 6-FAM/NED labeled primers in the presence of selected ddNTPs. At this step the positive reaction and control were mixed with the sequencing ladders and run on an Applied Biosystems 3130 capillary electrophoresis instrument. The raw capillary electrophoresis traces were processed using QuSHAPE software as described in (15). Probe-assisted secondary structure prediction was performed using RNAProbe webserver which allows automated normalization and analysis of RNA chemical probing results with various computational tools (10). Additionally, the base-pairing probabilities calculated with RNAfold were used to calculate the Shannon entropy for each nucleotide residue. Results are visualized in Figure 1 and Supplementary Figures S1–S5.

### Cryo-EM—sample freezing and data collection

Samples purified by SEC were applied to the grids in MCB buffer after RNA concentration adjustment. RNA purified from the gel was refolded by incubation in 80°C for 2 min in a buffer containing 20 mM HEPES pH 7.5, 50 mM KCl, 50 mM NaCl. After the denaturation step, the RNA was immediately placed on ice and MgCl_2_/DTT mixture was added to the final concentration of 1 mM. Samples (3 μl, 0.9 to 1.8 mg/ml) were applied to a glow-discharged (8 mA, 80 s) Quantifoil R2/2, 200 Cu mesh grid and vitrified in liquid ethane using a FEI Vitrobot Mark IV (Thermo Fisher Scientific) at 4°C with 95% humidity and 2 s blotting time. Data collection was performed using a Titan Krios G3i cryo-electron microscope (Thermo Fisher Scientific) at the SOLARIS National Synchrotron Radiation Centre (Krakow, Poland). The microscope operated at 300 kV was equipped with a BioQuantum energy filter (with a 20 eV energy slit) and a K3 camera (Gatan). Datasets of 4935 to 6699 movies were acquired in counting mode with a physical pixel size of 0.86 Å, 50 μm C2 condenser aperture and retracted objective aperture (Supplementary Table S2). The nominal defocus range was –2.7 μm to –0.9 μm with 0.3 μm increments (Supplementary Table S2). The total dose (fractionated into 40 frames) was 40 e/Å^2^ and the dose rate measured in vacuum was 16.4 e/pixel/s.

### Cryo-EM data processing

Cryo-EM images were processed using cryoSPARC 3.2 (16). Raw movies from the datasets (Supplementary Table S2 and Supplementary Figures S6–S9) were imported into cryoSPARC, motion corrected, and a contrast transfer function (CTF) was fitted. Initially, blob-picked particles were extracted with a box size of 256 pixels, subjected to two rounds of reference-free 2D classification. Selected particles from this process were used to generate *ab initio* volumes. Volumes displaying RNA secondary structures were subsequently selected. Corresponding 2D classes were low-pass filtered to 20 Å and utilized as templates for template picking. Template-picked particles were extracted with a box size of 256 pixels and subjected to two rounds of reference-free 2D classification. Selected particles from this stage were used to generate *ab initio* volumes. Only volumes exhibiting secondary structural elements were retained. The corresponding particles were re-extracted with a box size of 512 pixels and subjected to 3D variability analysis, 3D classification and 3D refinement. Particles that corresponded to minor variations in the longer stems impeded resolution improvement and were therefore excluded. Particles corresponding to the predominant class were utilized in the final refinement.

### Structural modeling in cryo-EM density maps

Previously, we used SimRNA (17) to build 3D models of SARS-CoV-2 structural elements, including SL5, using the chemical probing data available at the time of the analysis (18,19). Now, we also generated preliminary models of the SL5 elements from the A, C, and D lineages of βCoVs, using secondary structure restraints derived from the chemical probing data generated in this work. For each βCoV SL5 we identified 3D structure models with overall architectures compatible with the cryo-EM maps and fitted them to the respective cryo-EM densities using Chimera (20). We then refolded the initial models using SimRNA, optimizing their fit to the map using the cryo-EM density as a 3D restraint. For the best-fitted conformations from the refolding simulations, an ensemble of ten all-atom models was generated for each of the four RNAs, which were further refined against the map using the *real_space_refine* method from Phenix (21) and optimized using QRNAS (22). Restraints for the optimized models were generated using DoubleHelix (23) and used for one additional cycle of *real_space_refine*. The quality of the resulting models and ensembles was validated using MolProbity (24) and the fit of the models to the map was assessed using Q-score (25). Structure superposition and RMSD calculation were carried out with US-align (26). In the context of core junctions, two nucleotides located adjacent to the junction on each stem were incorporated, resulting in a total of 16 residues for the four-way junctions and 12 residues for the three-way junction. Images were rendered using UCSF Chimera (20) and Protein Imager (27).

### AFM air and liquid imaging

All RNA samples were diluted to a final concentration of 2 nM in RNAse-free 5 mM Tris pH 8.5 buffer. For air AFM imaging, we followed the sample preparation method described in (28). Briefly, we first pre-treated a freshly cleaved mica surface with 50 μl of 30 mM spermidine solution (Sigma–Aldrich) for 10 minutes followed by a wash step with 3 ml of Milli-Q water. 50 μl of 2 nM RNA sample was deposited onto the treated mica, incubated for one minute, and rinsed again with 3 ml of Milli-Q water. Finally, the sample was dried with a gentle stream of nitrogen gas and placed in the AFM for imaging.

For liquid AFM imaging, we followed the sample preparation method described in (29). 20 μl of adsorption buffer (5 mM Tris pH 8.5, 1 mM NiCl_2_) was deposited onto a freshly-cleaved mica sheet followed by addition of 20 μl of 2 nM RNA sample. After 10 min incubation, a further 30 μl of adsorption buffer and 50 μl of 5 mM Tris were added to the imaging chamber to a final volume of 120 μl.

AFM imaging was performed using a Cervantes Full mode AFM from Nanotec Electronica S.L. in tapping mode. For air imaging, we used PointProbePlus tips (PPPNCH Nanosensors) to collect images of 512 × 512 pixels from an area of 500 × 500 nm^2^. For liquid experiments, we used the 0.3 N/m cantilever with 90 kHz resonance frequency of the qp-BioAC probe (Nanosensors). Images of individual RNA molecules were 80 × 80 nm^2^ with a resolution of 3.2 pixel/nm.

### AFM image processing and volume map extraction

Raw images were plane corrected, and line flattened using the WSxM software (30). Individual molecules were then selected within an 80 nm square window, and the bicubic interpolation algorithm in MATLAB’s imresize was used to enlarge these molecules to 512 × 512 pixel^2^ images. This resizing method, previously used for AFM image analysis (31), improved domain selection by our image analysis algorithm. Using this method, our datasets were resampled to 1.56 Å per pixel. Our image analysis method is based on the approach presented by Gilmore *et al.* (28), where authors generated volume profiles along the main chain of the molecule and the cumulative volume in the profiles was used to estimate the number of nucleotides in each RNA region. This estimate was based on a conversion factor that relates the total volume of each molecule to the total number of nucleotides in the molecule. Although we used the basic volume-based approach, due to the size of our RNAs we replaced volume profiles with threshold-based boundary assignments. To implement this methodology, we developed an in-house MATLAB script to identify RNA domains and extract volumes. The data analysis workflow is as follows:

First, the volume of individual RNA molecules was determined as the volume of the AFM image above a certain threshold. This threshold was determined using the automated method described in (32) with its MATLAB implementation available at (https://www.mathworks.com/matlabcentral/fileexchange/3195-automatic-thresholding). The typical mean threshold value was 0.40 (0.10) nm [mean (SD)]. We also investigated other auto-thresholding methods (33,34), which gave comparable results (data not shown). In some cases, the final threshold required minor manual adjustments to maintain the integrity of the RNA chain.

RNA domain boundaries were then identified using a threshold-based approach. We hypothesized that higher regions in the image corresponded to structured segments made up of double-stranded RNA connected by ssRNA linkers. The initial molecule height threshold was incrementally increased until distinct regions emerged. This method produced between one and three thresholds depending on the morphology of the molecule. Once the regions were defined, the linkers were automatically selected by skeletonizing the molecule using MATLAB’s bwmorph ‘thin’ operation. Each domain and linker region were then labeled, extended to the threshold of the molecule, and its volume calculated using Eq. (1):


(1) \begin{equation*}\mathop \sum \limits_{i = 1}^M \mathop \sum \limits_{j = 1}^N \left( {\left( {{h}_{ij} - H} \right)\cdot{p}^2} \right)\end{equation*}

where the image area is *M* × *N* pixels squared, *p* is the pixel size in nm, ${h}_{ij}$ is the height in the pixel with coordinates (*i*, *j*) and *H* is the average background height.

Finally, our analysis provided volume measurements of different regions, which we presented as the normalized cumulative volume maps, similar to the method of Gilmore *et al.* (28). The estimation of sequence ranges was performed using a volume/nucleotide conversion factor (detailed in Supplementary Methods).

### Differential scanning fluorimetry thermal stability analysis

RNA samples were diluted to 200 nM and mixed with a 50x concentrated RiboGreen RNA fluorescent probe (Thermo Fisher Scientific) in 50 μl assay buffer containing 20 mM HEPES, pH 7.5, 5 mM DTT, 150 mM NaCl. Samples were incubated for 10 min at room temperature in the absence of light. 10 μl of sample was loaded in four repeats into the capillary (NanoTemper Technologies AN-041001). The end of the capillary was sealed with a liquid rubber seal (NanoTemper Technologies PR-P001). The capillaries were loaded into the Andromeda system from NanoTemper Technologies, to allow the acquisition of the fluorescence signal during the temperature gradient. Data were collected using AN.Control software v1.1 (NanoTemper Technologies AN-020001). The fluorescence excitation was adjusted to keep the maximum fluorescence intensity at 510 nm below 2000 counts. The temperature ramp was set at 1°C/min and the fluorescence signal was collected for the temperature range 25–95°C. Data with calculated inflection points corresponding to unfolding events were exported to xls file and processed using Origin Pro 2022 (OriginLabs). Signal processing included normalization against the amplitude of the fluorescence change and calculation of the first derivative of the fluorescence signal. The unfolding profiles and corresponding calculated Tms were plotted using Origin Pro 2022 (OriginLabs).

## Results

### Overall structure of the 5′-proximal regions in genomic RNAs of βCoV from A, B, C, and D subgenera

To identify the secondary structure elements within the 5′-proximal regions of the four βCoV genomic RNAs, we employed chemical probing on *in vitro* transcribed and refolded RNA fragments. We then performed experimentally guided modeling of the 5′-proximal regions of the four βCoVs using SHAPE-derived constraints, with DMS- and CMCT-derived data serving as orthogonal validation. The results for SARS-CoV-2 (nt 1–298) agreed well with previous studies (19,35,36), demonstrating our ability to accurately identify known structural elements, namely SL1–SL5 (Figure 1A). Despite the high degree of sequence divergence between the sequences of OC43-CoV (nt 1–368), MERS-CoV (nt 1–378), and RoBat-CoV (nt 1–265), we unambiguously identified the elements SL1, SL2, SL4, and SL5 – although SL3 was not detectable. These structural elements match regions of low Shannon entropy, indicating their structural stability, whereas linker regions are characterized by higher Shannon entropy values. SARS-CoV-2 transcriptional regulatory sequence (TRS-L) is located within the hairpin loop of SL3, whereas in other βCoV RNAs, it is a part of a flexible linker. A conserved 5′-CUUGY-3′ motif is located within SL2. Despite a lack of sequence conservation, size variations, a variable pattern of insertions/deletions and potential non-canonical base pairing, SL4 was identified in all four βCoV RNAs. In three subgenera, the SL5 region forms a four-way junction (4WJ) comprising the basal stem and three inner stems, namely SL5a, SL5b, and SL5c (Figure 1B). In subgenus D, SL5c is absent, causing SL5 to form a three-way junction (3WJ). The SL5a and SL5b loops of SARS-CoV-2, MERS-CoV, and RoBat-CoV share a common sequence motif (5′-UUYCGY-3′), whereas in RoBat-CoV, this motif is found in the SL4 loop and is completely absent in OC43-CoV.

### Cryo-electron microscopy unveils conserved 3D structures of junction motifs in the 5′-proximal regions of βCoVs from A, B, C, and D subgenera

**Figure 2.**
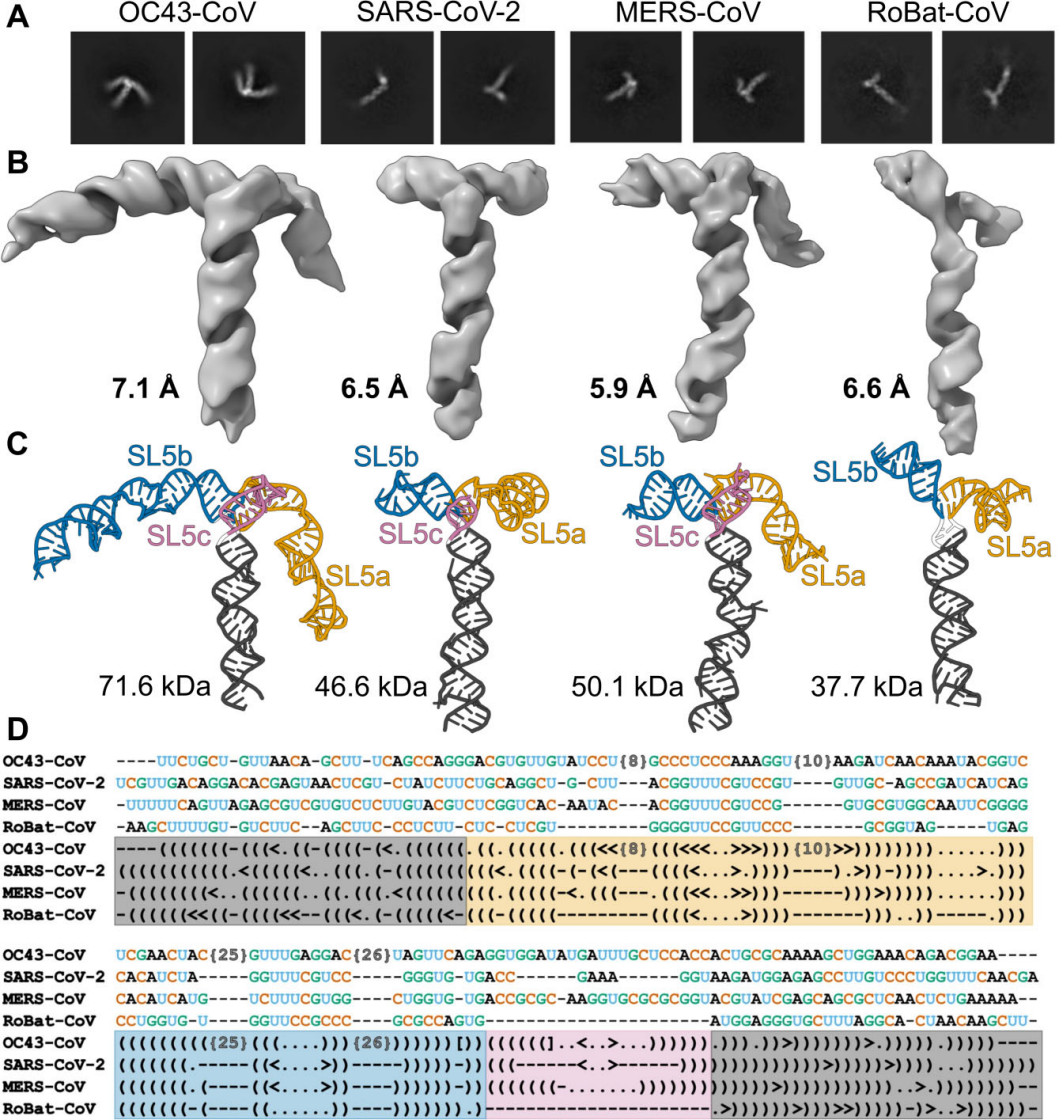
Structure conservation in the SL5 element in βCoVs: OC43-CoV, SARS-CoV-2, MERS-CoV, and RoBat-CoV. (**A**) Example reference-free 2D-class averages. (**B**) Cryo-EM maps. (**C**) Structural models. (**D**) Structure-based sequence alignment. Canonical and non-canonical base pairs are indicated by () and <> pairs, respectively, unpaired residues are indicated by dots, deletions are indicated by dashes. Significant insertions in OC43-CoV have been excluded for clarity and are indicated by the number of undisclosed residues in brackets {N}. Images rendered using Chimera (20) and Protein Imager (27).

Given the functional significance of the structural elements within the 5′-proximal regions of βCoVs, we sought to gain high-resolution structural insights into their SL5 elements. We were particularly interested in the most complex structural elements from all four viruses that yielded homogenous and properly folded particles on vitrified electron microscope grids. Cryo-EM single particle analyses generated high-quality 2D class averages (Figure 2A) and maps at 7.1, 6.5, 5.9, 6.6 Å overall resolutions for OC43-CoV, SARS-CoV-2, MERS-CoV, and RoBat-CoV, respectively (Figure 2B). During the 3D reconstruction and refinement steps, particles with slightly varied conformations of the helical stems were observed. These differences were minimal and reflected the intrinsic RNA flexibility. Only particles corresponding to the predominant class were used for the final refinement resulting in one final map for each viral RNA.

We constructed atomic models for the four maps obtained by a combination of manual model building and flexible refinement with 3D restraints on the cryo-EM maps and secondary structure restraints based on the chemical probing data. The modeling process yielded ensembles comprising 10 structures for each of the four RNAs (Supplementary Figure S10). The average root mean square deviation (RMSD) within each ensemble was 0.91, 0.74, 0.82, and 0.64 Å, respectively. The quality of the stereochemistry of models was validated using MolProbity (24) and their fit to the Coulomb density map was evaluated using Q-score (25) (Supplementary Table S2). At 5.9–7.1 Å estimated accuracy, individual atomic positions and non-canonical base pairs cannot be confidently assigned. Nevertheless, the backbone conformation and the approximate position of base pairs and individual bases can be confidently traced. All four structures exhibited a T-like shape, with the topology of the junctions featuring the SL5 basal stem positioned almost at right angles to the coaxial stack of SL5a/SL5b stems. In the three structures with SL5c, this stem was also co-axially stacked with the SL5 stem. Variations of the shape were mainly due to the lengths of the SL5a, SL5b, and SL5c arms (Figure 2C). SL5a and SL5b are the longest in OC43-CoV.

**Figure 3.**
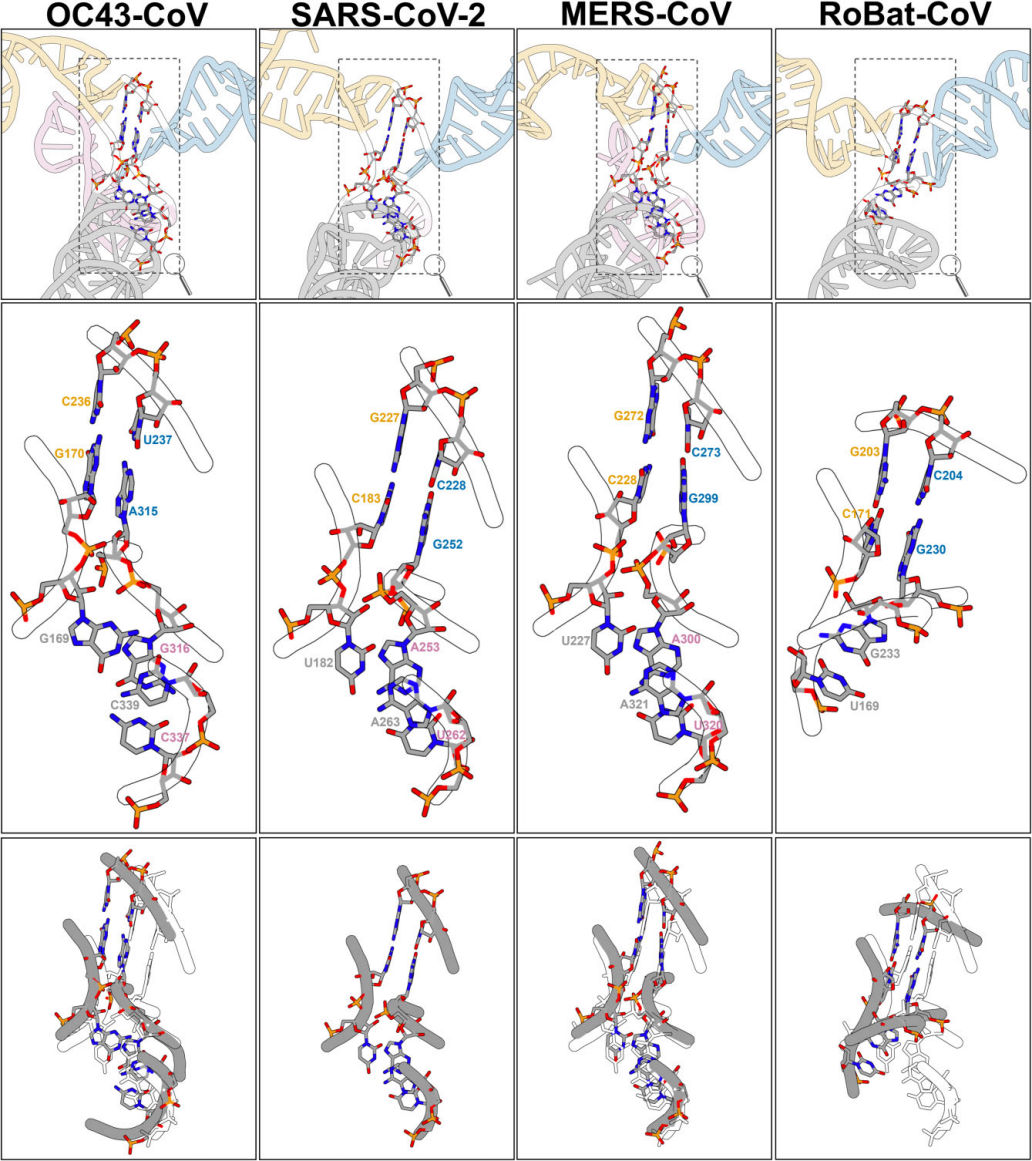
SL5 junction comparison. Stems are distinguished by colors: SL5 basal stem in grey, SL5a in yellow, SL5b in blue, and SL5c in pink. Upper panel: SL5 cartoon representation and junction residues in all-atom representation. Middle panel: SL5 junctions in all atom representation. Lower panel: OC43-CoV, MERS-CoV, and RoBat-CoV SL5 junctions (in grey) superimposed on the SARS-CoV-2 SL5 junction (in white).

Comparison of the models of the four SL5s revealed a striking similarity of the junction core. The SL5 4WJ in OC43-CoV, SARS-CoV-2, MERS-CoV was formed by four canonical base pairs, coming from the four stems, while in RoBat-CoV the SL5 3WJ was formed by two canonical pairs and one G–U wobble pair (Supplementary Figure S11). In SARS-CoV-2 and MERS-CoV RNAs, the base pairs at the junction motif were the same. In all four structures, the coaxial stack of stems SL5a/SL5b was continuous with no unpaired nucleotides between them. In OC43-CoV RNA, the junction had one unpaired nucleotide (A338) between the SL5 basal stem and SL5c. Nevertheless, the overall structure of the junction remained the same as in SARS-CoV-2 and MERS-CoV. In RoBat-CoV RNA, unpaired nucleotides were present between the SL5 basal stem and SL5a (U170) and between the SL5 basal stem and SL5b (A231, U232). The superposition of the junction core of the four SL5 models indicated conserved geometry (Figure 3). An overview of all superimposition RMSD values is presented in Supplementary Table S5. The junctions of SARS-CoV-2 and MERS-CoV were most similar to each other, with an RMSD of 2.36 Å (Supplementary Table S5). The greatest differences were observed between RoBat-CoV 3WJ and the three 4WJs, with the highest RMSD being 3.69 Å when compared with the OC43-CoV junction. This is attributed to the absence of SL5c in RoBat-CoV RNA, causing the basal SL5 to differ more significantly compared to the SL5 4WJs. Nevertheless, the absence of SL5c in RoBat-CoV RNA had only a minor effect on the overall SL5 geometry (Figure 2C).

The structure-based sequence alignment revealed only traces of sequence similarity, limited to the hairpin loops in SL5a and SL5b, which do not interact with each other in the 3D structures. Even the pattern of canonical base pairs is not conserved in the stems, as all four stems exhibit different non-canonical pairs with multiple short insertions and deletions (Figure 2D). Overall, the SL5 elements exhibited remarkable global structure conservation, despite the divergence at the level of individual nucleotide residues and their base pairs.

### Air AFM reveals flexible connections between structured domains within the 5′-proximal regions of different βCoVs

**Figure 4.**
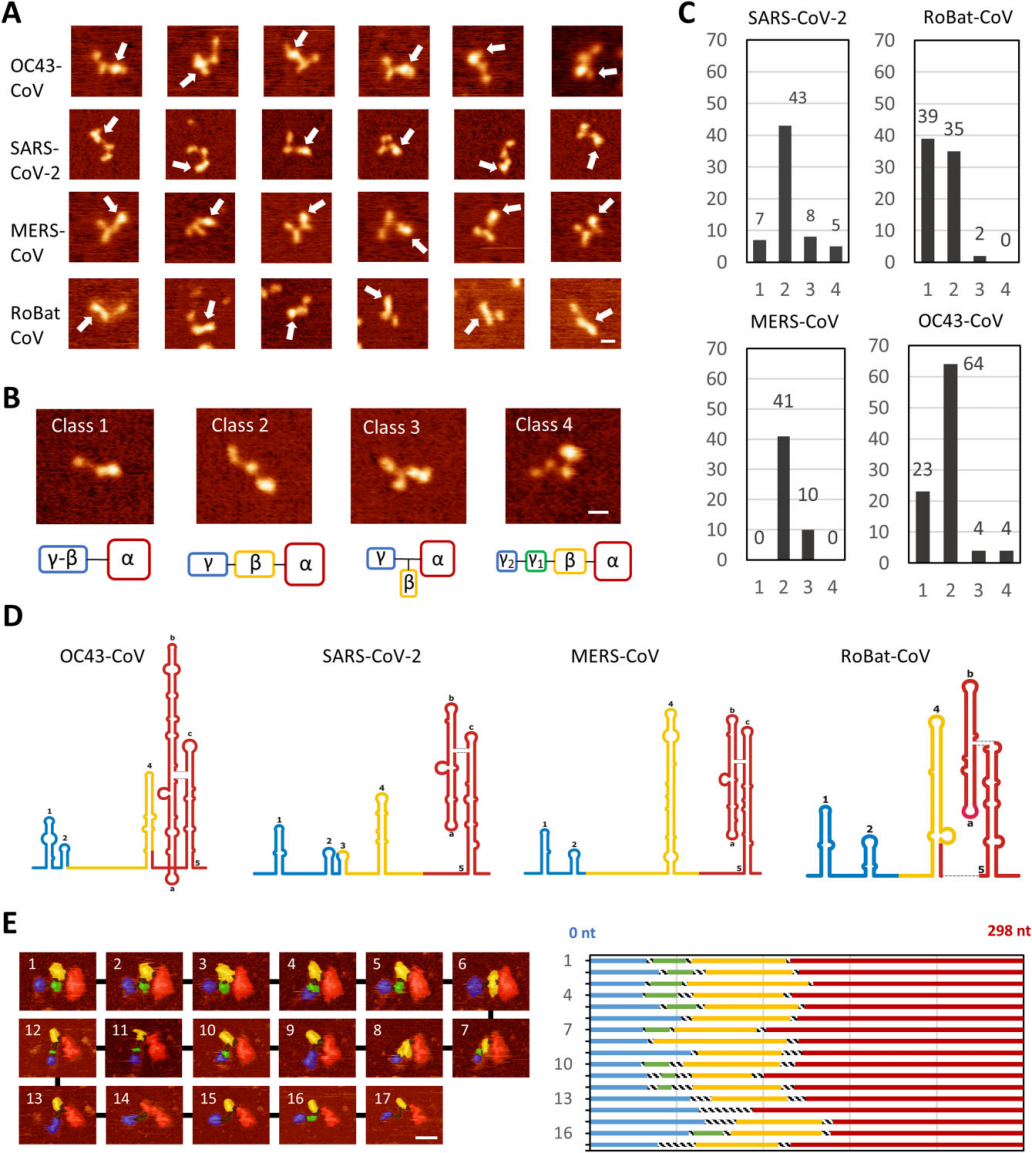
AFM analysis of the 5′-proximal regions of genomic RNAs from different βCoVs. (**A**) Representative air AFM images of individual RNA molecules for the four βCoVs analyzed in this study. White arrows indicate the brightest region. Noticeable conformational differences were observed between samples. (**B**) Representative examples of the four classes of molecules observed, categorized by their region arrangement. The α-domain is assigned to the brightest region, likely representing the SL5 element. (**C**) Distribution of molecules in different classes of domain arrangement. (**D**) Volume analysis of the images provides an estimate of the nucleotide sequence range covered by each region. Average nucleotide ranges for each sample resulted in the three regions (Supplementary Figure S15, Supplementary Table S3). (**E**) Liquid AFM imaging captured domain rearrangements. A series of consecutive images of the SARS-CoV-2 RNA serves as a representative example (Supplementary Video S1). Volume analysis indicates rearrangements of the SL2 and SL3 elements. The bar size in all images is 16 nm. The color gradient, from dark to bright, spans 1.5 nm in air AFM images and 3.5 nm in liquid AFM images. Additional liquid AFM imaging of other βCoV RNAs is shown in Supplementary Figure S16.

Next, we used atomic force microscopy (AFM) to image the complete 5′-proximal regions of genomic RNAs from βCoVs OC43-CoV, SARS-CoV-2, MERS-CoV, and RoBat-CoV (Figure 4A). AFM images were obtained under air conditions by adsorption of the viral RNA in the absence of Mg^2+^ ions (see Materials and methods). This adsorption technique was shown to be efficient for observing the secondary structure of RNA molecules (28,37). Each sample produced a homogeneous population of molecules, with an average volume roughly proportional to the sequence length (Supplementary Figures S12 and S13).

Molecules exhibited different conformations usually composed of two, three or four distinct regions, which appeared as globules in AFM images. Interestingly, a brighter region located at one edge of the molecule was clearly identified in most of the AFM images (Figure 4A). As outlined in Materials and methods, the observed structures of all molecules were considered as a series of blob-like structures connected by thin linkers. A height threshold was employed manually to define domains and linkers. The structures were categorized into four classes as illustrated in Figure 4B, with each domain designated by Greek letters, starting from the brightest (largest) blob labelled as α. Class 1 corresponds to an arrangement of two regions. Class 2 corresponds to an arrangement of three consecutive regions. Class 3 corresponds to an arrangement of three regions but with a bifurcation. Class 4 corresponds to an arrangement of four regions (Figure 4B). Class 2 was most common in three βCoVs (80% in MERS-CoV, 68% in SARS-CoV-2, 67% in OC43-CoV), whereas almost equal populations of Class 1 and Class 2 were found in RoBat-CoV (51% and 46%, respectively). Class 1 was also relatively frequent in OC43-CoV. Class 3 was the third, minor arrangement detected for all four RNAs. Class 4 was a very rare arrangement, detected only in SARS-CoV-2 and OC43-CoV (Figure 4C).

To gain insight into the internal distribution of the structural elements within each molecule, we extracted volume maps based on heights, which provide more detailed quantitative information. Using our image analysis protocol (see Materials and methods for details), we measured the volume of the structured regions in the 5′-proximal regions of the four βCoV RNAs and estimated the sequence range corresponding to each structural element. These sequence ranges matched well to the respective structural elements determined by chemical probing (Supplementary Table S3, Supplementary Figure S14). To calculate an average estimate of the sequence range across the four classes of RNA, all sequence ranges were combined resulting in a bimodal distribution across all four βCoV (Supplementary Figure S15). By applying a Gaussian fit to each peak, we identified the three average nucleotide sequence ranges, which were then mapped onto the secondary structure representation of the four fragments (Figure 4D). A striking correlation was found between the sequence ranges extracted for each region and the corresponding structural elements identified in βCoV RNAs. Importantly, the largest region was confidently assigned to SL5, the most prominent structured element present in all four viral RNAs. Moreover, the structured regions showed variations in size and shape among the βCoV RNAs, consistent with the different sizes of their respective structural elements, such as the largest and Y-shaped SL5 region in OC43-CoV, and the more compact and elongated SL5 in RoBat-CoV. For MERS-CoV, a rod-like appearance was observed in the middle region, consistent with the very long SL4 element. In all βCoVs a blob-like feature was observed in the smallest γ domain, consistent with a bundle of the short hairpin-loop structures SL1 and SL2.

### AFM in solution reveals a dynamic arrangement of structured elements and flexible linkers of βCoVs RNAs

We first imaged the 5′-proximal region of SARS-CoV-2 in solution. The RNA molecules were adsorbed using a low concentration of NiCl_2_ (see Materials and methods), which allowed the molecules some mobility. In addition, this approach facilitated a clearer distinction between the double-stranded and single-stranded regions. The movement of structured elements was captured by comparing successive images, highlighting greater flexibility in the linker regions. Detailed analysis of one movie (Figure 4E) showed that the SL5 region had limited mobility, whereas the rest of the RNA, with its smaller structural elements, displayed a more dynamic movement (Supplementary Video S1). Volume maps of the molecule across all frames revealed that the SL2/SL3 region (corresponding to the smallest structural feature of the molecule) was occasionally visible. This suggests that the 5′-proximal region of the SARS-CoV-2 RNA genome adopts a dynamic structure, with SL5 as the main stable element. However, due to resolution limitations, we could not always distinguish the smallest domains separately.

Next, we visualized the 5′-proximal regions of the MERS-CoV, RoBat-CoV, and OC43-CoV (Supplementary Figure S16, Supplementary Videos S4–S6). Similarly to SARS-CoV-2, the use of liquid AFM imaging enabled better discrimination between individual structured domains, (brighter regions) interconnected by linkers revealing a dynamically organized structure for all βCoVs. Occasionally, melting of a structured region was observed (Supplementary Figure S17, Supplementary Video S2 and S3). This was probably caused by the punctual application of excessive force by the AFM tip. In SARS-CoV-2 RNA, the original four-blob structure underwent a rearrangement, resulting in two individual blobs connected by a single-stranded RNA. In OC43-CoV RNA, the SL1 element eventually unfolded. Together, these data support the hypothesis that the blob-like structures observed in AFM represent structured RNA domains. The flexible nature of the linkers connecting the structured elements allowed for various configurations of the entire 5′-proximal region, which may be relevant for facilitating a variety of interactions. Importantly, we never observed tip-induced disassembly of SL5 in the βCoV samples, suggesting a greater stability compared to the other structural elements (SL1–4).

### Thermal stability of the 5′-proximal region and the SL5 element in βCoVs RNAs

We analyzed the thermal stability of the 5′-proximal regions in βCoV RNAs to better understand the dynamics of the folded regions and their propensity to undergo large conformational changes. Among three βCoV RNAs, the average melting temperature (Tm) for the 5′-proximal region was found to be in a very narrow range from 60.4°C to 62.4°C. (Supplementary Table S4). We also analyzed the isolated SL5 elements, and the resulting Tm values were similar to their longer counterparts, ranging from 60.9°C for MERS-CoV to 62.1°C for SARS-CoV. For these RNAs, the difference in Tm between the complete 5′-proximal region and the isolated SL5 element was less than 1°C. However, RoBat-CoV stood out with higher average Tm values in both the 5′-proximal region (66.24°C) and the SL5 element (63.60°C). Notably, only for RoBat-CoV the Tm of the 5′-proximal region and the SL5 element differ by more than 2.5°C, indicating that the SL5 element of this representative exhibits lower thermal stability compared to the entire 5′-proximal region.

**Figure 5.**
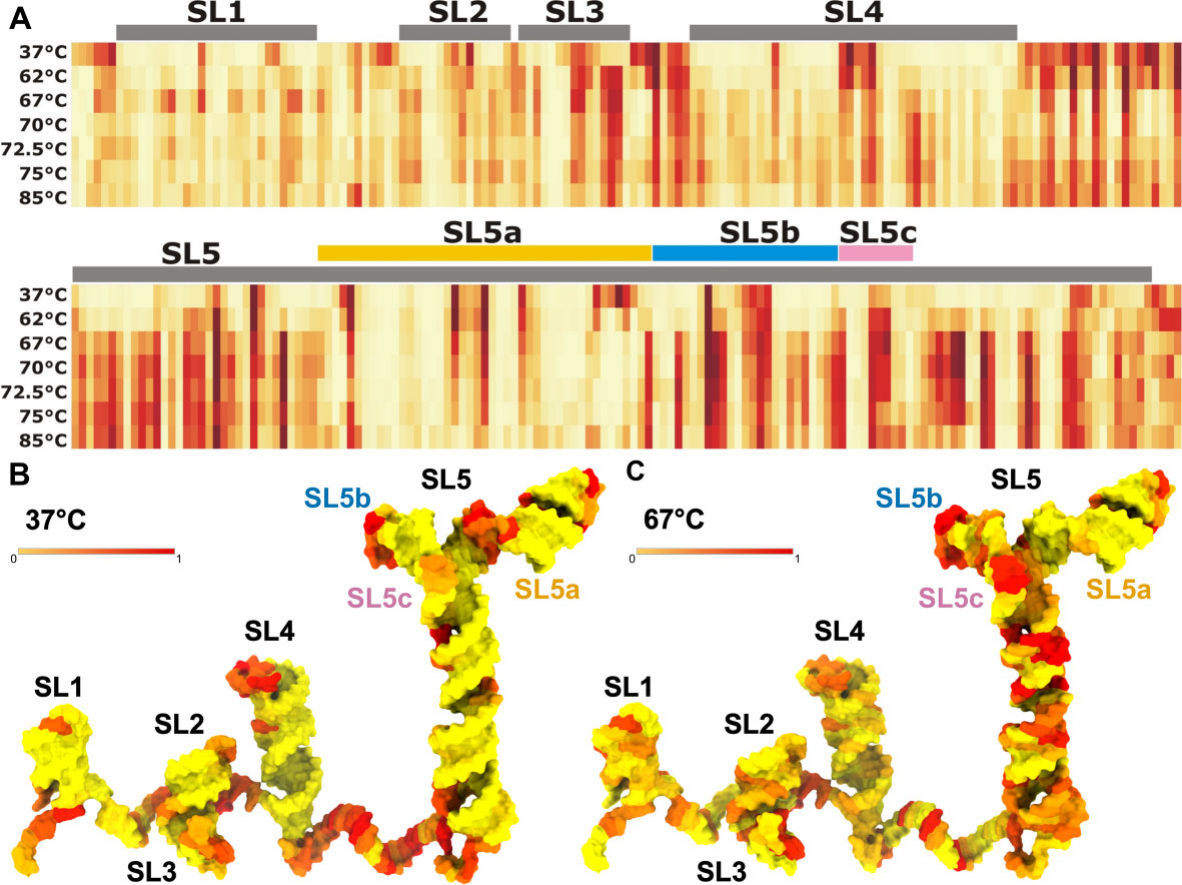
Thermal stability of the 5′-proximal region of SARS-CoV-2 as probed by SHAPE. (**A**) Heatmap of SHAPE reactivity level variations when probed in the 37–85°C temperature range. (**B**) SARS-CoV-2 5′-proximal region model colored according to SHAPE reactivity at 37°C. (**C**) A model of the SARS-CoV-2 5′-proximal region model 3D structure colored according to SHAPE reactivity at 67°C. Individual structural elements SL1–SL5 were arranged spatially to facilitate visualization in 2D (detailed in Supplementary Methods). Detailed values of reactivities are shown as bar plots in Supplementary Figure S18.

To identify sites in the 5′-proximal region of SARS-CoV-2 susceptible to temperature-induced structural changes chemical probing was performed using SHAPE analysis at different temperatures. Analysis of the overall reactivity patterns revealed distinct differences corresponding to secondary and tertiary interactions and transitions as a function of temperature (Figure 5A and Supplementary Figure S18). At 37°C, the SHAPE experiment accurately captured the structure of the 5′-proximal region of SARS-CoV-2, demonstrating stable secondary and tertiary interactions. However, as the temperature increased, the transcript exhibited a greater tendency to form 2′-*O*-adducts, indicating increased local nucleotide flexibility in the RNA.

Significant variations in the reactivity pattern were observed at 62°C and higher temperatures (Figure 5A and Supplementary Figure S18). In particular, the SL3 stem, which overlaps the TRS-L, displayed significant changes in reactivity, suggesting that it is the least stable structural element within the 5′-proximal region. Within the SL5 element, the first increases in reactivity values were also observed at 62°C in the basal SL5 stem, particularly for nucleotides 165–170, located close to one of the bulges, and nucleotides 265–269, encompassing the ORF1ab AUG start codon. The alterations in reactivity values along the 5′-proximal region observed at temperatures of 67°C and above followed the ascending trend observed at 62°C with only a few cases of reactivity decrease (SL4 loop; SL5a loop nucleotides U201 and U205; SL5 bulge nucleotides 220–225).

## Discussion

The 5′-proximal regions of βCoV RNA genomes play a crucial role in translation, replication, and genome packaging (5). Complexes of 400 nt long 5′-termini of the viral RNA together with nucleocapsid proteins can form virion-like particles (38), indicating dynamic properties vital for functional virion packing. Our study delves into the unique features and variations among different members of the βCoV genus. Using a combination of chemical probing, AFM, and cryoEM, we have gained insights into previously unknown 3D structures and their dynamics.

In particular, our study revealed significant conservation in key structural features of the 5′-proximal regions of RNA genomes across four βCoV subgenera. They all share structural elements SL1, SL2, SL4, and SL5, despite high sequence variability and variable patterns of base pairs and bulges. SARS-CoV-2 is distinguished from other coronaviruses by the presence of the SL3 structural element. In SARS-CoV-2, TRS-L is within the SL3 hairpin loop, while in other βCoV RNAs, it is in linker regions with high reactivity in chemical probing experiments. SL3 is thought to have a transient stem-loop conformation (5,7,35,36,39,40), confirmed by our melting tests where SL3 disrupted early. TRS-L’s location or its thermal sensitivity might relate to its role in genome transcription. A previous study found that the TRS and the SL4-following linker are targets for the nucleocapsid protein's N-terminal domain (39), with structured elements such as SL2 and SL4 aiding this binding. SL5 is the largest and most complex structural element in the 5′-proximal regions of βCoV RNA genomes. Our results provided detailed insights into the SL5 3D structure, revealing a 4WJ architecture in OC43-CoV, SARS-CoV-2, and MERS-CoV, and a 3WJ in RoBat-CoV. While recent research on HCoV-OC43’s 5′-proximal region suggested a dynamic SL5 structure with up to six stem-loops (41), this prediction was made only for the 329 5′-terminal nucleotides. Our analysis showed that further 35 nucleotides are essential for the formation of a complete SL5. Contrary to the dynamic model of HCoV-OC43 SL5, we propose a stable SL5 structure with a 4WJ, akin to other βCoVs.

Sequence motifs in βCoV genomes differ in location and quantity. The GHGUG motif, recognized by the host protein RBM24, is a case in point. In SARS-CoV-2, RBM24 binding to the motif in the SL4 stem hinders 80S ribosome assembly, limiting mRNA translation (42). Coronaviruses possess one (like MERS-CoV) or two (as in SARS-CoV-2 and RoBat-CoV) copies of this motif, situated within structural elements. In MERS-CoV, the GCGUG motif is located in the SL5a, near the loop. In SARS-CoV-2, only the SL4 GUGUG motif binds RBM24, not the SL5b version (42). The last guanine (G252) of that motif in SL5b contributes to a four-way junction (see Figure 3 and Supplementary Figure S11). The SL5 stable 4WJ structure might prevent RBM24 binding. However, it is important to note that in the case of the murine βCoV RNA (43), the presence of the GHGUG motif within SL4 was not essential for the viral RNA replication.

The 5′-proximal regions of βCoV RNA genomes show consistent structural dynamics, as evidenced by our AFM experiments. Air AFM measurements offer direct visualization of structural elements in these regions, confirming the conservation of their architecture. Multiple conformers can be imaged simultaneously, revealing structural variability, as demonstrated in (44). The dynamics of the 5′-proximal regions are evident in the similar conformations adopted on the surface. Compact regions are linked by flexible single-stranded linkers, leading to a pliable configuration. The conformations visualized for all four βCoVs were categorized into four groups based on the arrangement of two, three, or four regions. All four viral RNAs exhibited a large structured domain attributed to the SL5 region consistent with chemical probing and cryo-EM analyses. This consistency of assignment was observed for most structural elements. Structural elements under 40 nucleotides are hard to resolve individually with AFM, and our imaging combined SL1 and SL2 into one structural entity. Distinct SL5 shapes were identified, such as the Y-shape in OC43-CoV, the more compact blob-like shape in MERS-CoV and SARS-CoV-2, and the rod-like shape in RoBat-CoV, in strong agreement with the cryo-EM maps of SL5. The AFM measurements of the 5′-proximal region of the four βCoVs RNAs in solution provided additional insights into structural dynamics and flexibility. The motion captured in AFM movies, even though time resolution was limited in our setup, showed a stable behavior of SL5 and the flexibility of other structures, suggesting the 4WJ ensures stability.

Our study on the thermal stability of the 5′-proximal regions showed variations in Tm among the coronaviral RNAs. MERS-CoV had the lowest Tm, while RoBat-CoV had the highest, likely due to its fewer single-stranded regions. The stability measurements, AFM analyses, and SHAPE probing resulted in highly complementary results, showing that the SL5 structure is vital for the stability of the 5′-proximal region in βCoV RNAs. Monitoring the SL5 melting temperature could be an efficient method to identify molecules that destabilize its conserved structure, paving the way to target it with small molecules. The Tm of SL5 was consistent with that of the 5′-proximal regions for most samples. In a chemical probing of SARS-CoV-2 RNA at varied temperatures, base pairs near the AUG start codon in the SL5 basal stem broke first, highlighting the role of dynamic regions in translation regulation.

The chemical probing, AFM image analysis, cryo-EM data of structured domains, and thermal stability analysis, together provide valuable insights into global conformation and dynamics of the 5′-proximal region of βCoV RNA genomes. Chemical probing provides sequence-level information and allows to identify structured regions and flexible linkers. Cryo-EM excels at resolving well-structured regions like SL5, providing structural details. AFM, a non-invasive visualization method for RNA, avoids procedures like cryogenic freezing. Instead, it relies solely on surface adsorption. Its strength lies in visualizing larger and flexible RNA molecules, which are hard to characterize at high resolution with cryo-EM. Single-stranded RNA studies using AFM are still relatively scarce due to difficulty in sample preparation and image analysis. Previous works mainly imaged large viral RNA molecules (37,45) and lncRNA (46,47) to determine global architectures. Our work can be treated as a guide for AFM image analysis supported by other experimental analyses, which can account for small domains of around 50 nucleotides.

Our interdisciplinary study on the 3D structures and dynamics of the 5′-proximal region of βCoV RNA genomes provides a foundational resource for researchers aiming to target the viral RNAs for therapeutic interventions. The conserved structural elements may have potential as targets for antiviral strategies. The SL5 element, with its substructures that unfold in high-temperature, is a notable target candidate. Understanding the structural context of conserved sequence motifs interacting with host proteins can deepen knowledge of host-virus interactions. As the global scientific community confronts the challenges of diseases caused by βCoVs, our findings contribute to the development of informed, structure-based approaches to combat these pathogens.

## Supplementary Material

gkae144_Supplemental_Files

## Data Availability

The coordinates for all models in this work have been deposited in the RCSB PDB database under the PDB IDs 8QO2, 8QO5, 8QO4, and 8QO3. The final refined maps for all structures in this work have been deposited in the EMDB under the EMD IDs EMD-18520, EMD-18523, EMD-18522, and EMD-18521.
