# Supplementary material for: Conserved structures and dynamics in 5′-proximal regions of Betacoronavirus RNA genomes
Source: Nucleic Acids Res. 2024 Mar 1;52(6):3419–32. doi: 10.1093/nar/gkae144 (PMC11014237; doi:10.1093/nar/gkae144)
Supplement: gkae144_Supplemental_Files [file gkae144_supplemental_files.zip › BetaCoV_5UTR_SI_revised.docx]

**SUPPLEMENTARY INFORMATION**

**Conserved Structures and Dynamics in 5′-Proximal Regions of Betacoronavirus RNA Genomes**

Tales Rocha de Moura^1,†^, Elżbieta Purta^1,†^, Agata Bernat^1,†^, Eva M. Martin-Cuevas^2,†^, Małgorzata Kurkowska^1^, Eugene F. Baulin^1^, Sunandan Mukherjee^1^, Jakub Nowak^3^, Artur P. Biela^3^, Michal Rawski^3,4^, Sebastian Glatt^3^, Fernando Moreno Herrero^2^, Janusz M. Bujnicki^1,*​^

ORCID numbers:

Tales Rocha de Moura: 0000-0001-5492-8419

Agata Bernat: 0000-0002-5555-9464

Elżbieta Purta: 0000-0003-0960-548X

Eva Martín Cuevas: 0000-0002-8976-9478

Eugene Baulin: 0000-0003-4694-9783

Sunandan Mukherjee: 0000-0002-4361-0103

Sebastian Glatt: 0000-0003-2815-7133

Fernando Moreno-Herrero: 0000-0003-4083-1709

Janusz M. Bujnicki: 0000-0002-6633-165X

^1^ Laboratory of Bioinformatics and Protein Engineering, International Institute of Molecular and Cell Biology in Warsaw, ul. Ks. Trojdena 4, 02-109 Warsaw, Poland

^2^ Department of Macromolecular Structures, Centro Nacional de Biotecnología, Consejo Superior de Investigaciones Científicas, Madrid, Spain

^3^ Malopolska Centre of Biotechnology, Jagiellonian University, Krakow, Poland

^4^ National Synchrotron Radiation Centre SOLARIS, Jagiellonian University, Krakow, Poland;

^*^ To whom correspondence should be addressed. Email: [janusz@iimcb.gov.pl](mailto:janusz@iimcb.gov.pl)

^†^ The authors wish it to be known that, in their opinion, the first four authors should be regarded as Joint First Authors.

**Keywords:** RNA structure, 5′-proximal region, Coronavirus, Cryo-EM

# Authors’ Contributions

J.M.B., T.R.M., E.P., A.B., M.K. conceived and designed the analysis; E.P., A.B., M.K. prepared RNAs for all types of experiments presented in the paper and performed chemical probing experiments and analyses; M.R. collected cryo-EM data and initially curated as well as analyzed the cryo-EM datasets with the support of A.P.B. and S.G.; T.R.M. processed the cryo-EM data and built the 3D models with the help of E.B. and S.M.; E.M.M.-C. performed all AFM experiments and analyses of AFM data, including the development of specific methods for AFM sample absorption and analysis. F.M.-H. supervised AFM data acquisition and analysis. E.B. analyzed the sequence data and generated alignments; J.N. determined the thermal stability of the RNA samples and analyzed the data with the support of S.G.; J.M.B., T.R.M., E.P., A.B. drafted the manuscript; all authors edited the manuscript and approved its final form.

**Supplementary Methods**

Determination of domains distributions from volume maps.

To illustrate the image analysis methodology, we considered the SARS-CoV-2 case. We averaged volume values of domains belonging to the same class (Supplementary Fig. 14). This provided a sequence range which corresponds to the structured parts of the molecule. Among the different classes considered, Class 2 fits well to the sequence range given by the volume analysis with the SL5 element marked in blue, the adjacent domain in yellow and the end domain in red.

The 3 remaining classes can be interpreted as the following: Class 1 presents the SL5 junction and only one adjacent domain which is split into two smaller domains in Class 2, most likely comprising SL1, SL2, SL3, and SL4. In Class 3, the volume of linkers from both sides of SL4 is included in the middle region, resulting in only three nucleotide ranges. Last, Class 4 presents SL2 and SL3 as one separated structural element.

Generation of the SARS-CoV-2 RNA 3D model for the visualization of the probing data.

Based on the experimental structural information obtained in this work and other experimental analyses of individual structural elements (1–6) we generated a tentative 3D model of the 5′-proximal region in SARS-CoV-2 genomic RNA using ModeRNA (7). The secondary structure elements were manually arranged in two dimensions to create a flattened representation, thereby enhancing the visualization of chemical probing data on the 3D structure. The model of the entire 5′-proximal region is meant for visualization only and does not represent any specific biologically relevant conformation of this RNA.

**GenBank accession numbers of sequences used in the alignment:**

subgenus A: KM349744.1, NC_046954.1, JN874562.1, LC061274.1, MH687968.1, KY370049.1, KY370048.1, KY370051.1, KY370043.1, NC_006577, AF207902.1, AF029248.1, OK073091.1,

subgenus B: KP886809.1, MK062184.1, KF569996.1, MT040336.1, KY352407.1, MG772934.1, KF636752.1, HQ166910.1, MN908947.3

subgenus C: KT368871.1, KC869678.4, KJ473821.1, MG987421.1, MG021451.1, MG021452.1, KC545386.1, MK907287.1, EF065507.1, NC_009020.1, NC_019843.3

subgenus D: MK492263.1, EF065515.1, NC_009021.1, MK492263.1, NC_030886.1


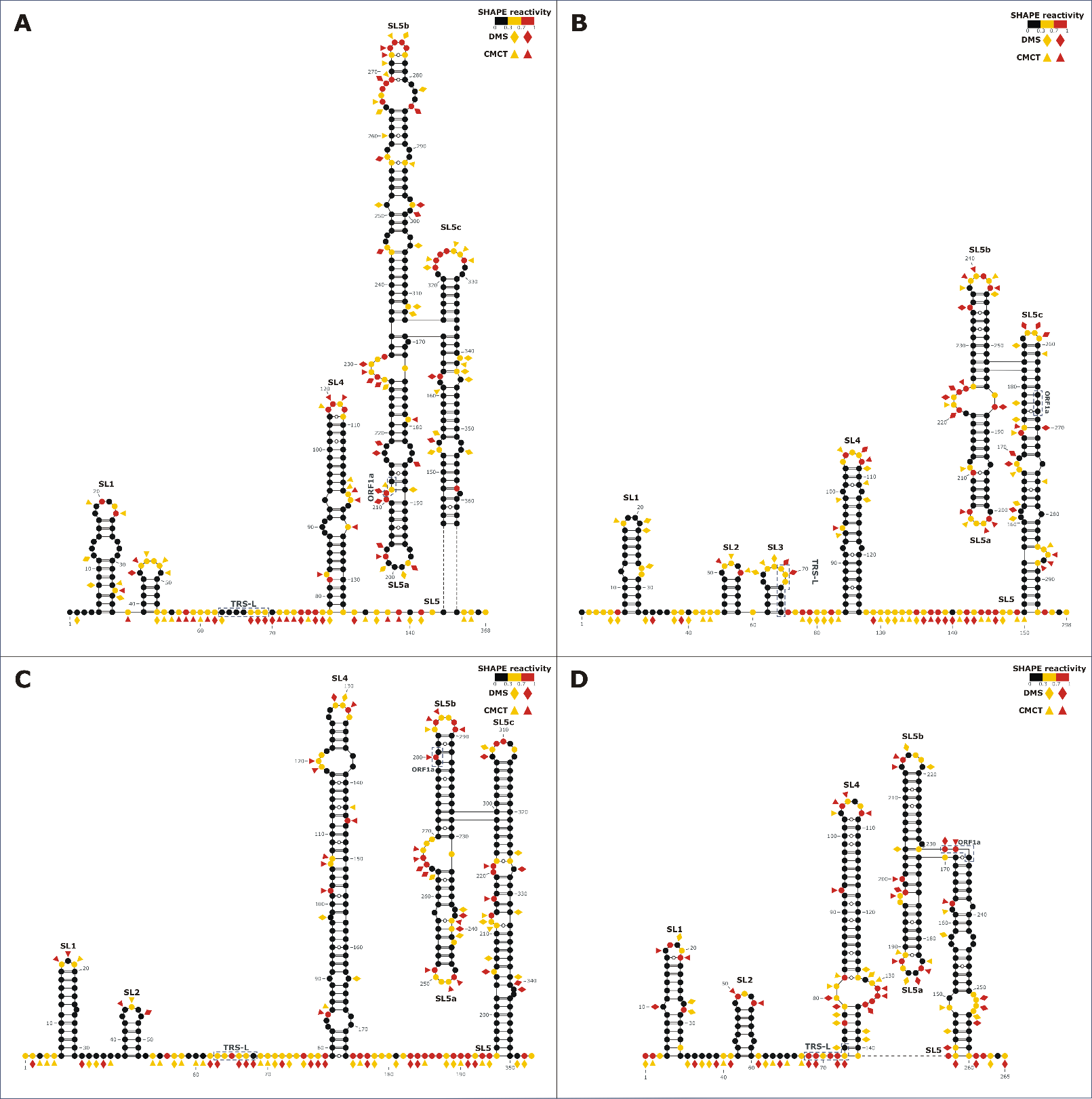
**Supplementary Figure 1: Secondary structure probing reactivities.** The probing reactivities are superimposed on the 5′-proximal region structures of OC43-CoV (A), SARS-CoV-2 (B), MERS-CoV (C), RoBat-CoV (D). Normalized reactivities across three biological replicates for the in vitro probing experiments are presented. Highly (red) and moderately (yellow) reactive residues from in vitro SHAPE (circles) and DMS (diamonds) and CMCT (triangles) experiments are indicated. Highlighted features include stem-loops 1-5 (SL1 – SL5), the leader transcriptional regulatory sequence TRS-L and the start codon of ORF1a. Substructures within SL5 are labeled as a, b, c. In SL5, the individual base pairs were manually edited for positioning them according to the 3D models.


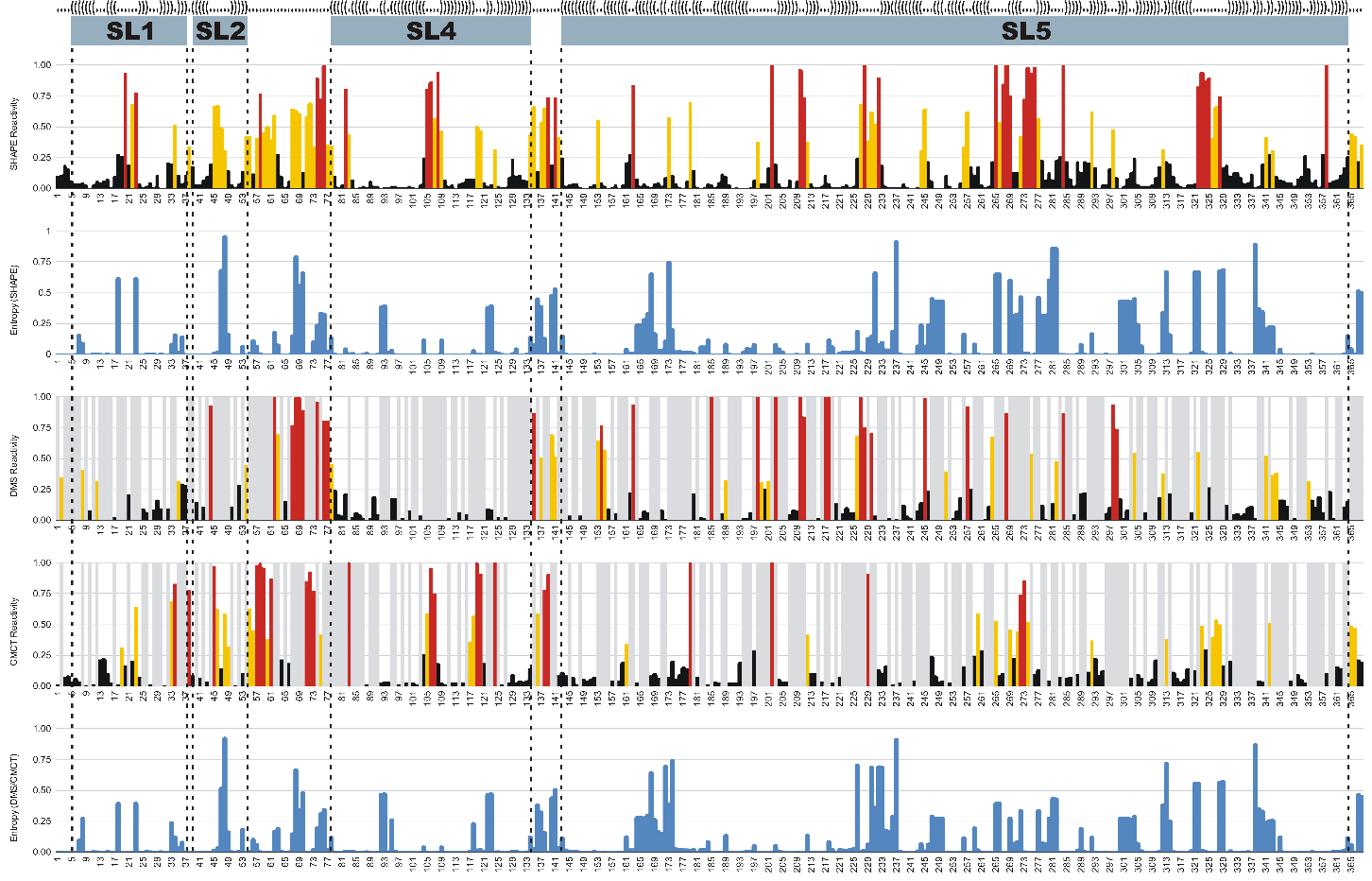
**Supplementary Figure 2:** **Reactivity plots for SHAPE, DMS and CMCT probing of OC43.** Shannon entropies were calculated for SHAPE and for combined data of DMS and CMCT.


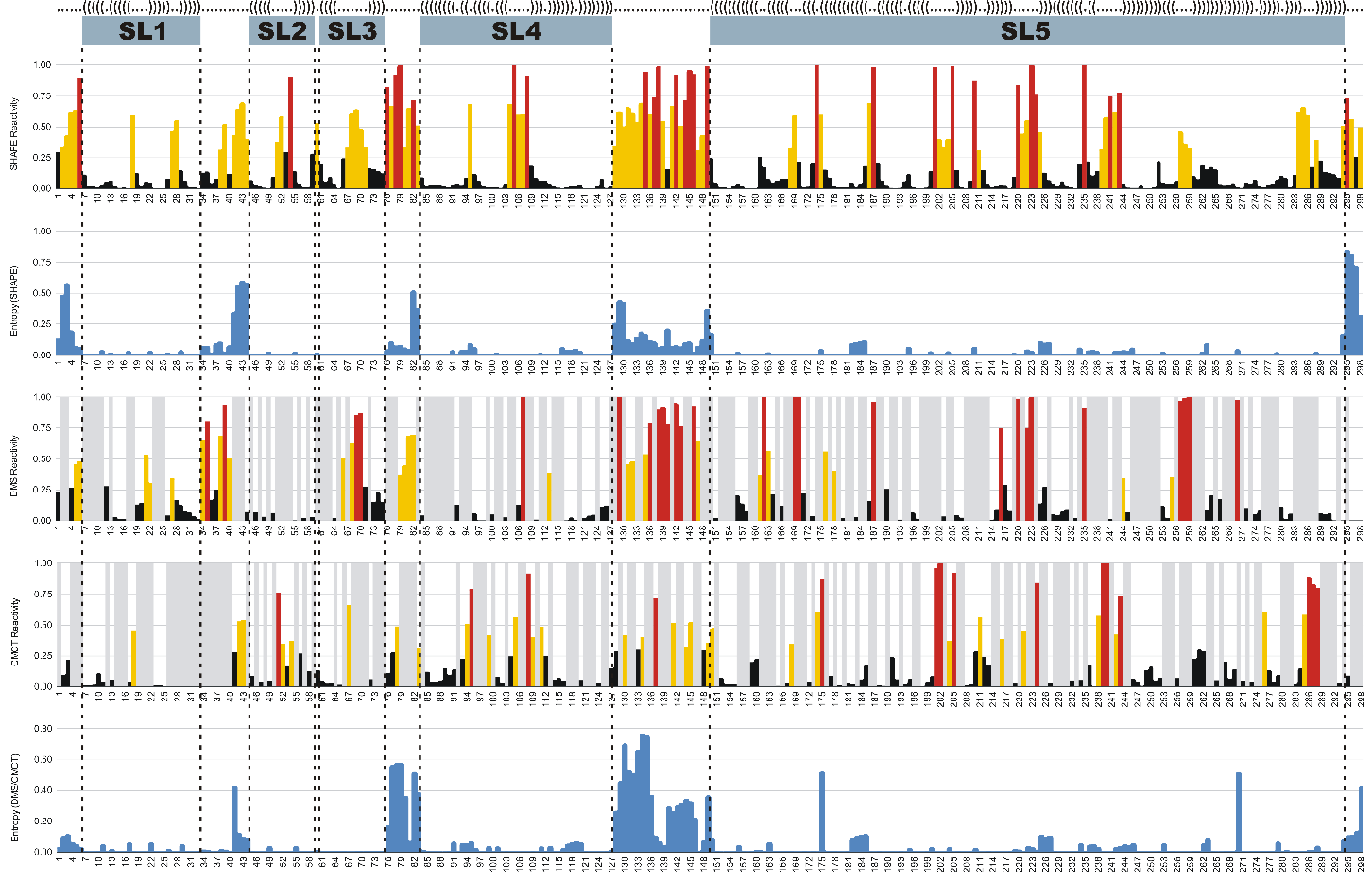
**Supplementary Figure 3:** **Reactivity plots for SHAPE, DMS and CMCT probing of SARS-CoV-2.** Shannon entropies were calculated for SHAPE and for combined data of DMS and CMCT.


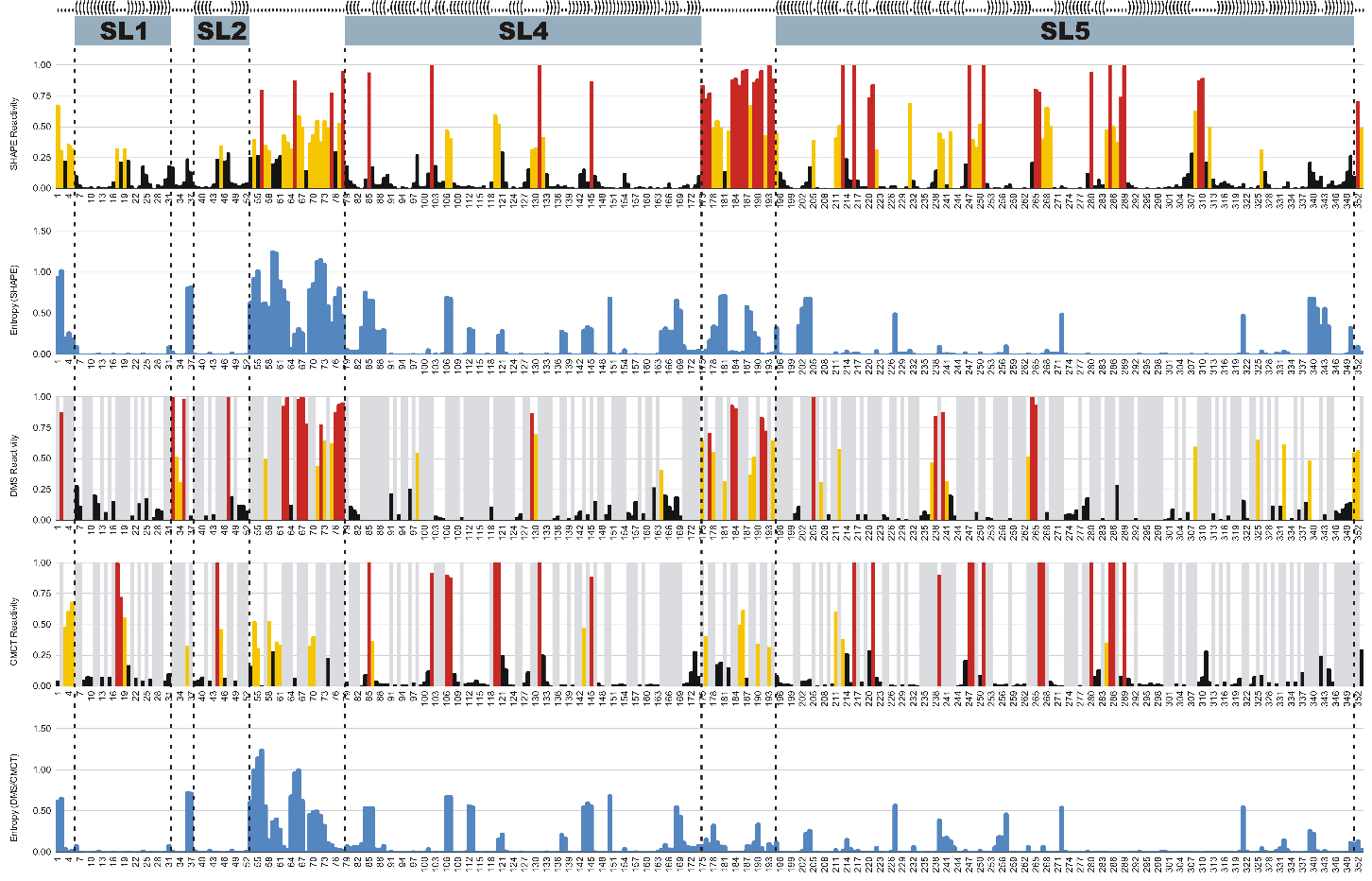
**Supplementary Figure 4:** **Reactivity plots for SHAPE, DMS and CMCT probing of MERS-CoV.** Shannon entropies were calculated for SHAPE and for combined data of DMS and CMCT.


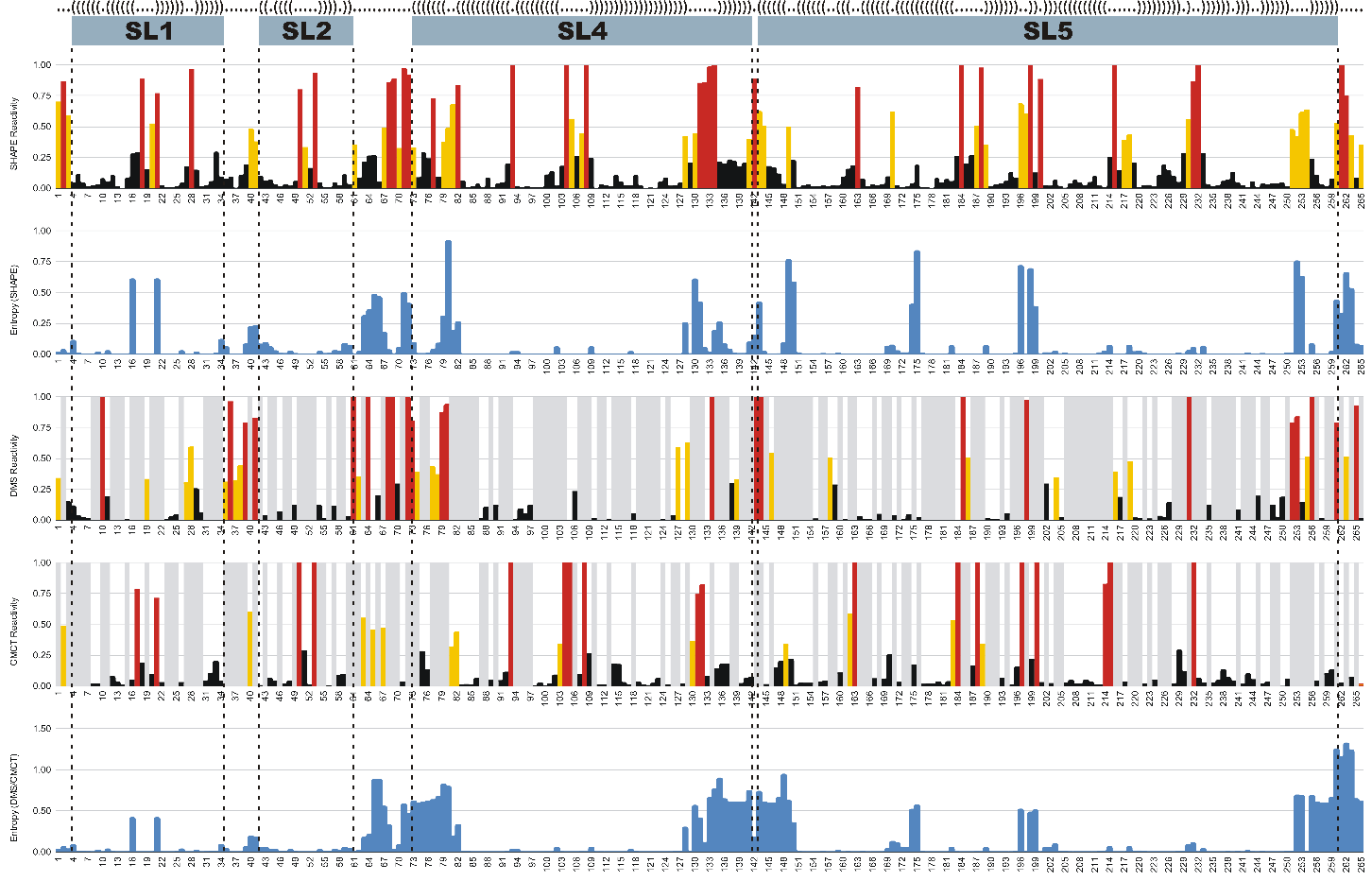
**Supplementary Figure 5:** **Reactivity plots for SHAPE, DMS and CMCT probing of RoBat-CoV.** Shannon entropies were calculated for SHAPE and for combined data of DMS and CMCT.


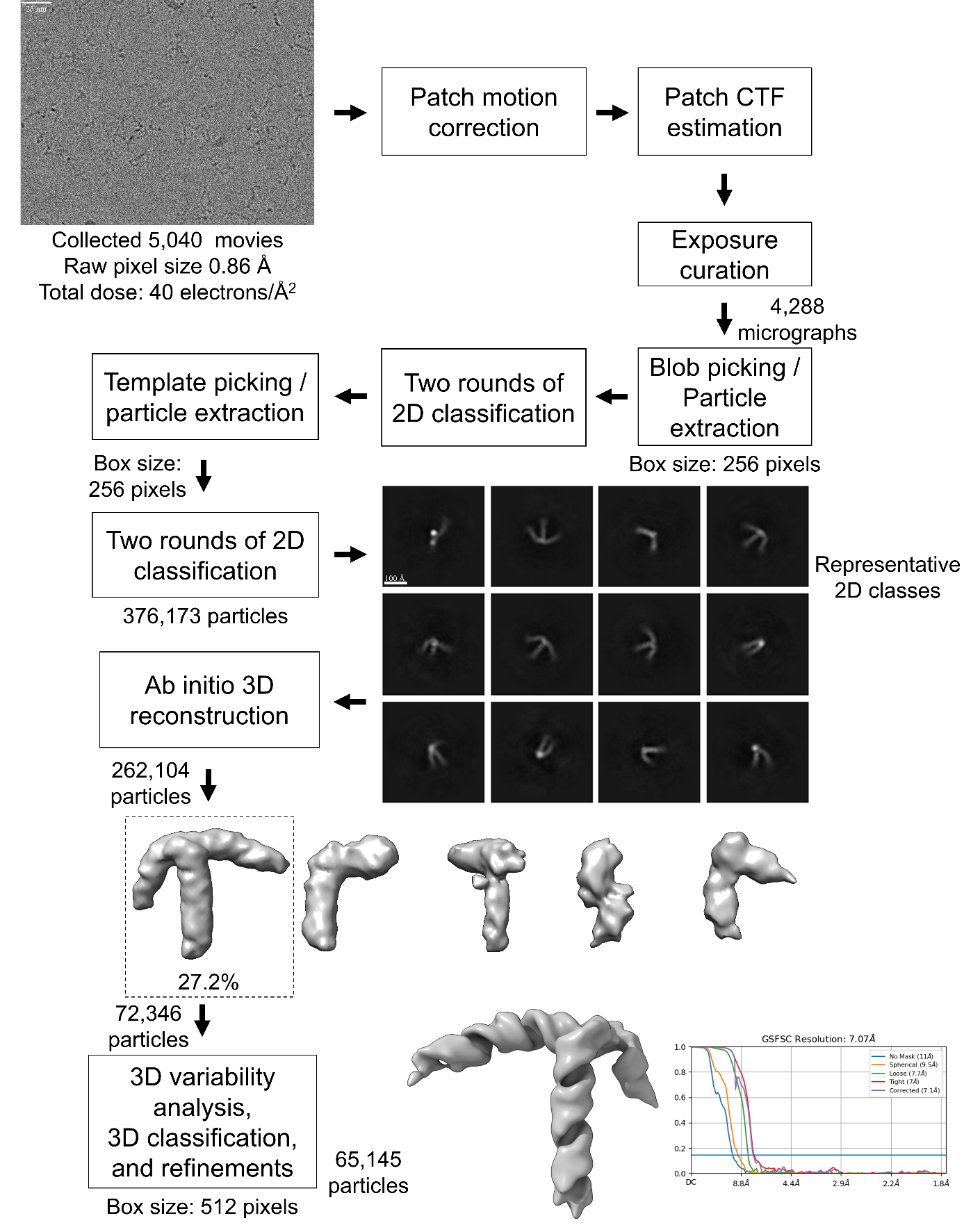


**Supplementary Figure 6: Cryo-EM processing OC43-CoV workflow.** Representative micrograph of vitrified OC43-CoV, (scale bar 25 nm). Representative 2D classes (scale bar 100 Å). Global resolution estimate determined using the Gold-standard FSC approach.


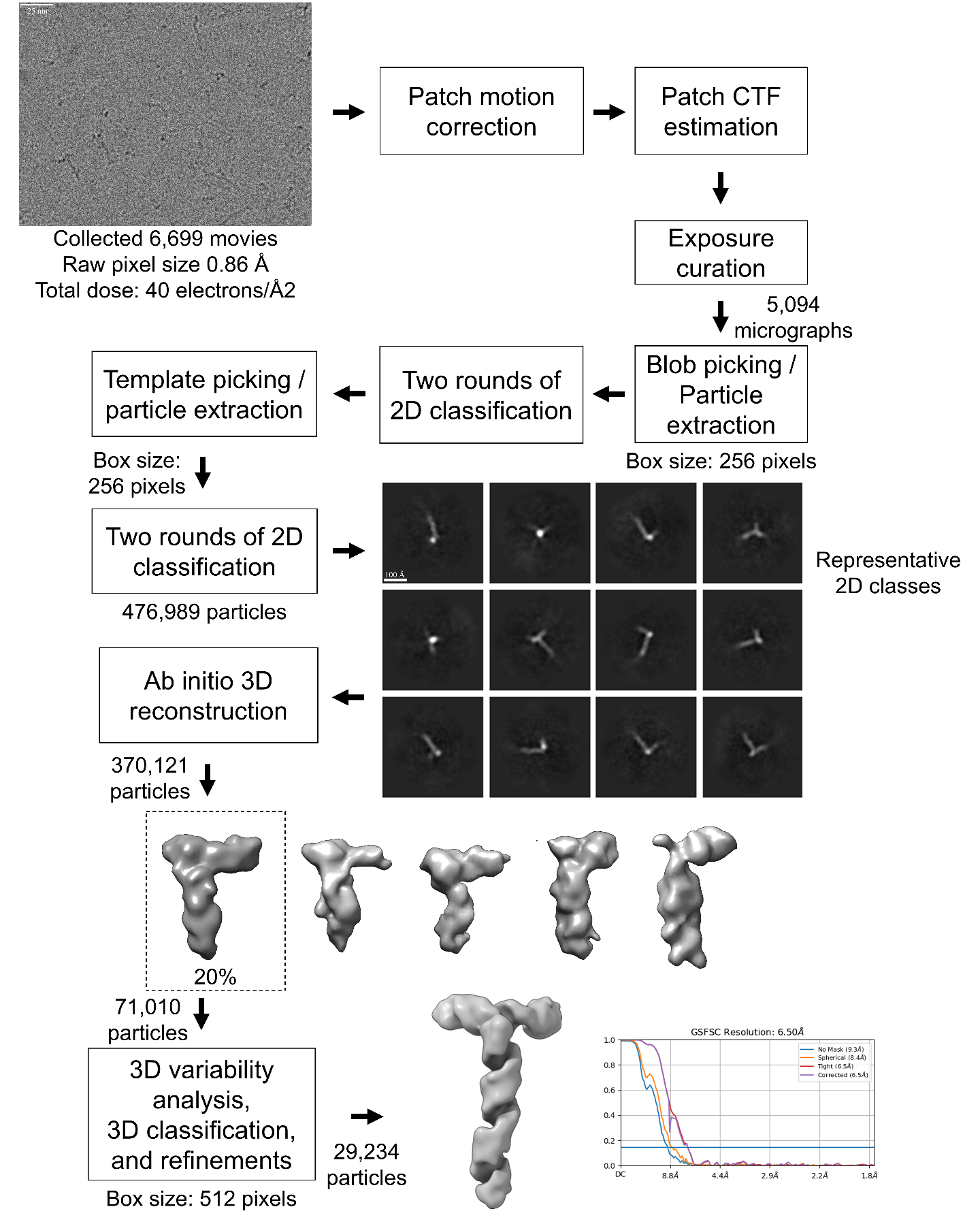
**Supplementary Figure 7: Cryo-EM processing SARS-CoV-2 workflow.** Representative micrograph of vitrified SARS-CoV-2, (scale bar 25 nm). Representative 2D classes (scale bar 100 Å). Global resolution estimate determined using the Gold-standard FSC approach.
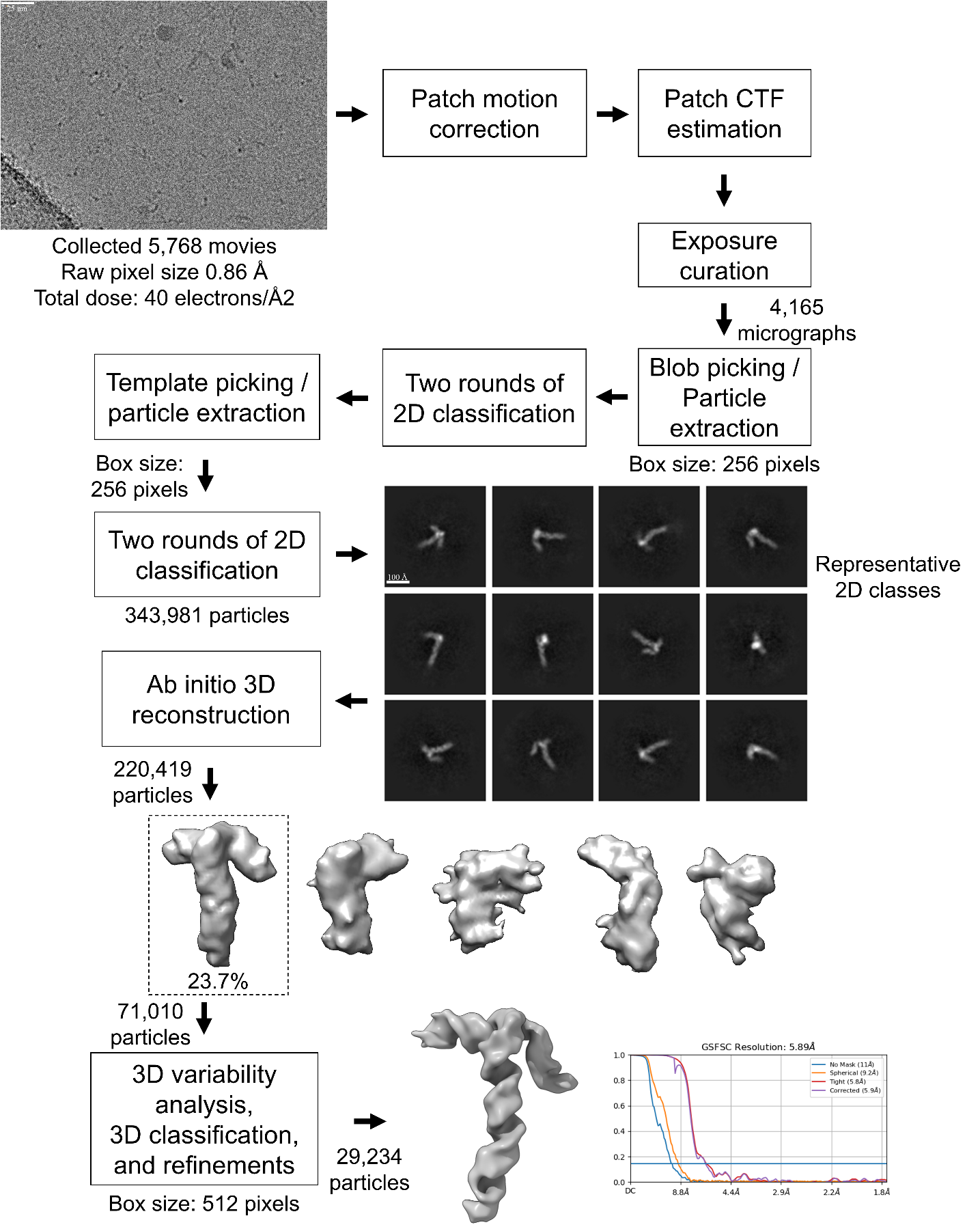
 **Supplementary Figure 8: Cryo-EM processing MERS-CoV workflow.** Representative micrograph of vitrified MERS-CoV, (scale bar 25 nm). Representative 2D classes (scale bar 100 Å). Global resolution estimate determined using the Gold-standard FSC approach.
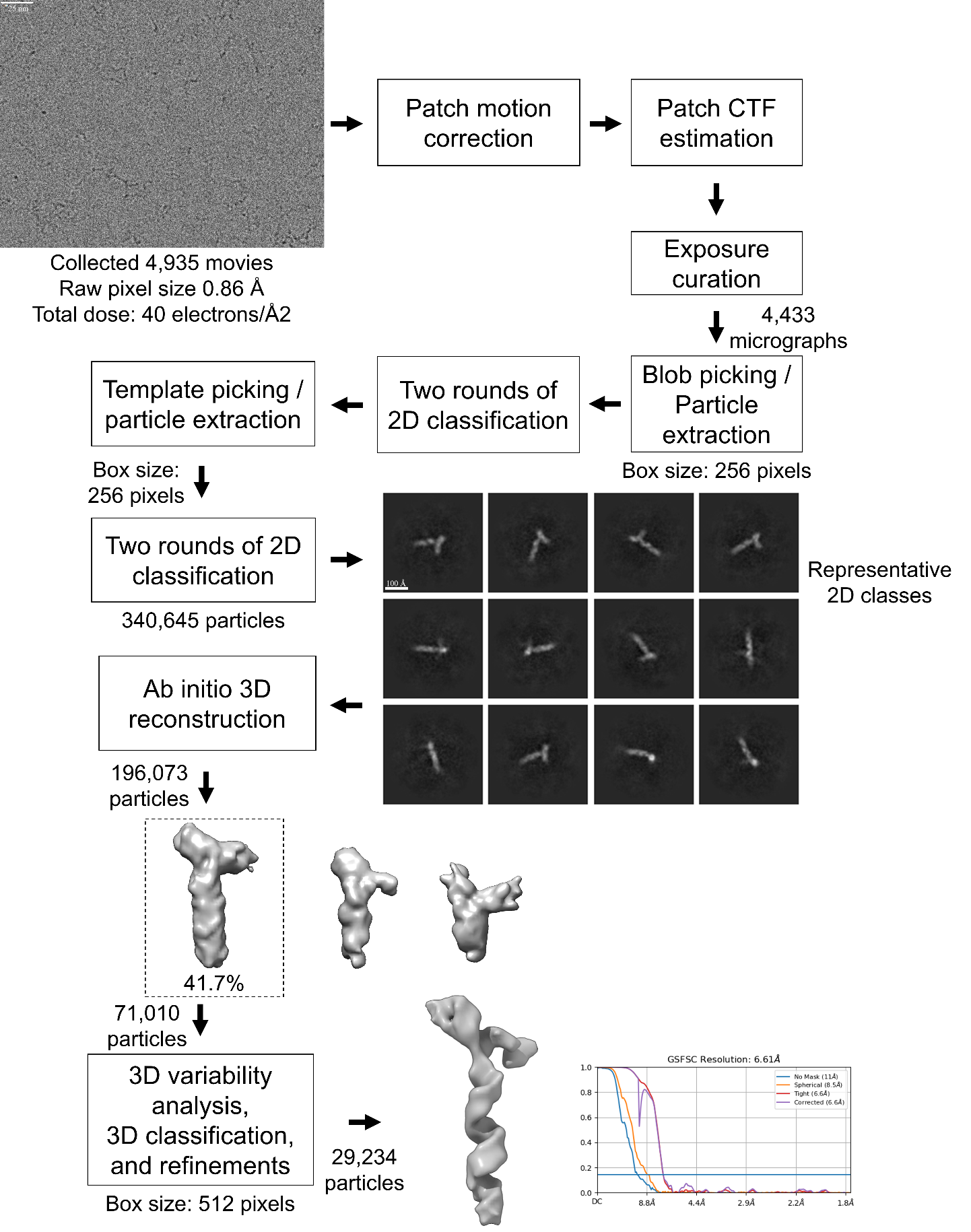
**Supplementary Figure 9: Cryo-EM processing RoBat-CoV workflow.** Representative micrograph of vitrified RoBat-CoV, (scale bar 25 nm). Representative 2D classes (scale bar 100 Å). Global resolution estimate determined using the Gold-standard FSC approach.


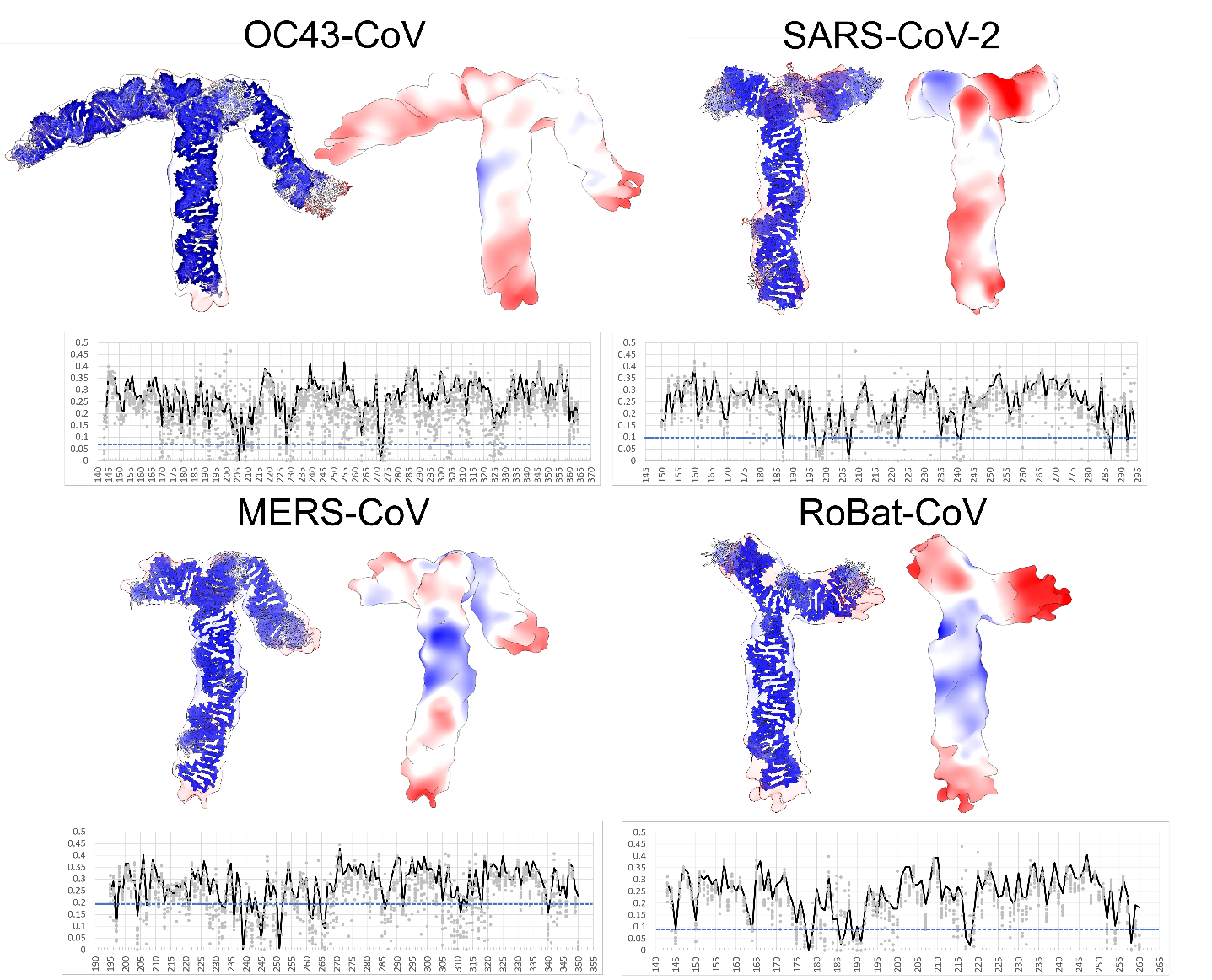
**Supplementary Figure 10: Ensemble models built into experimental density maps.** Ten SL5 junction models colored based on RMSD, and corresponding density maps colored according to the local resolution (blue to red, lower to higher values). Q-score plots for the ten ensemble models, grey dots for individual residue values, black line for average values, and dotted blue line for expected Q-score value of the corresponding resolution (7.1 Å, 6.5 Å, 5.9 Å, 6.6 Å for OC43-CoV, SARS-CoV-2, MERS-CoV, and RoBat-CoV, respectively).


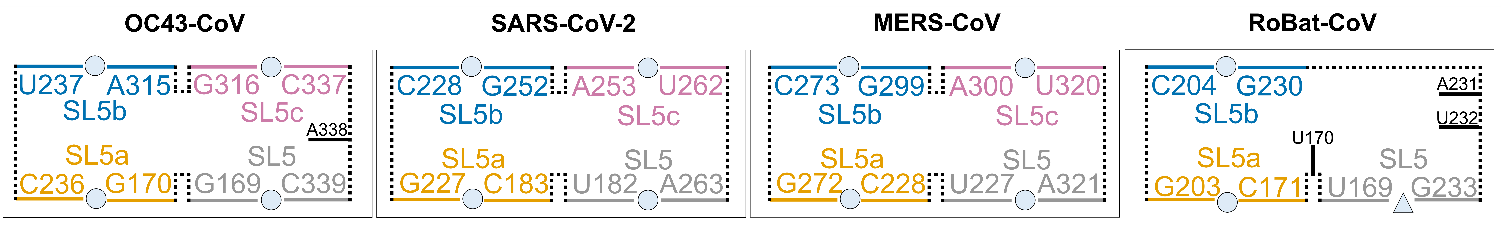
**Supplementary Figure 11: Comparison of SL5 junctions in a schematic representation.** Circles indicate canonical base pairs, triangles indicate a wobble pair, unpaired residues are in black.


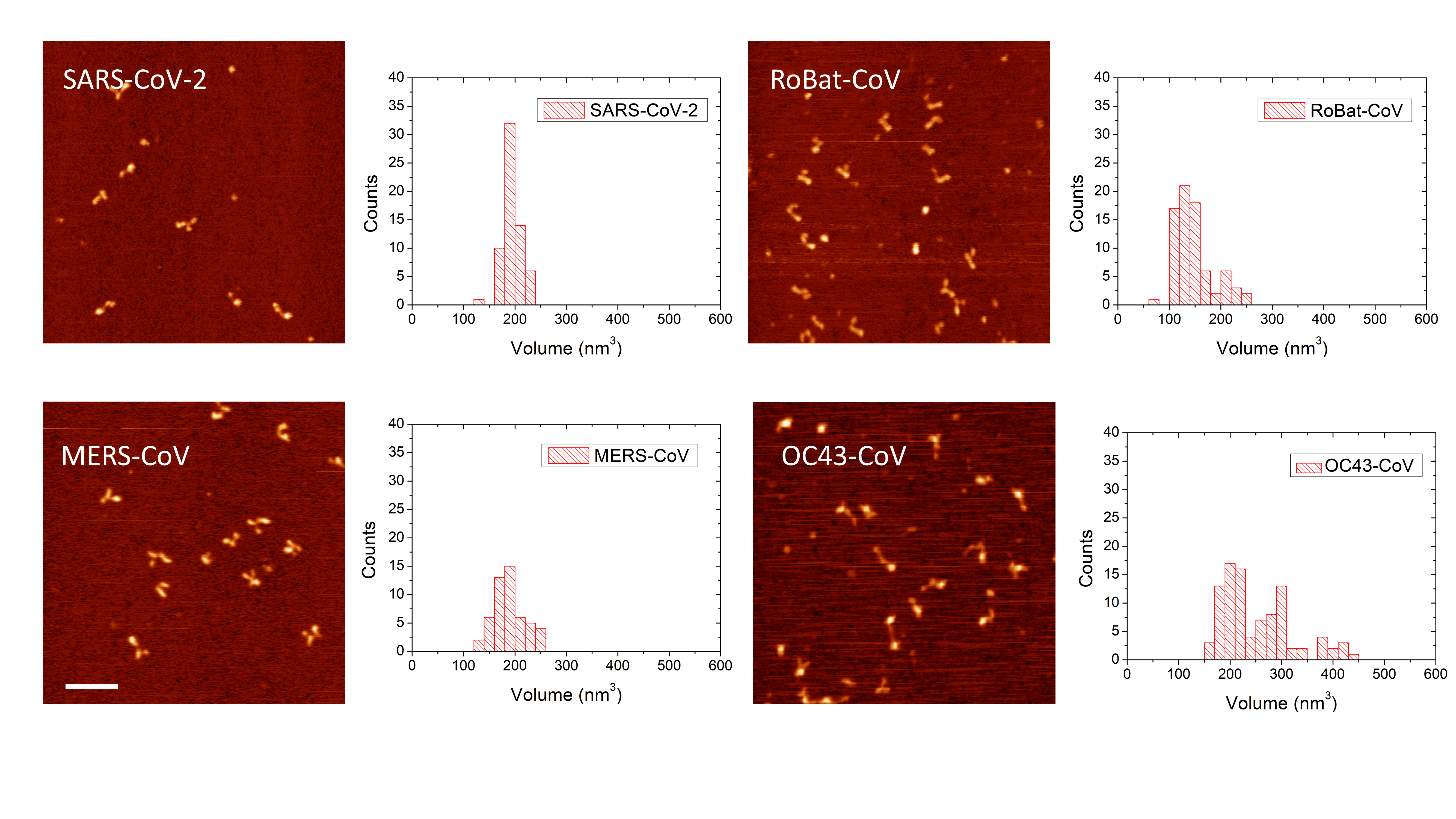


**Supplementary Figure 12:** **Representative AFM images and volume histograms of each sample.** The SARS-CoV-2 sample shows a volume distribution of 194 ± 2 nm^3^ with N = 63 molecules (mean ± standard error of the mean = SD/$\sqrt{N}$) yielding a very homogeneous population. The Robat-CoV and OC43-CoV showed a wider volume distribution (148 ± 4 nm^3^ N = 76 and 251 ± 7 nm^3^ N = 95, respectively). The MERS-CoV sample showed a similar deviation (190 ± 4 nm^3^ N = 51). Bin width of 20 nm^3^.


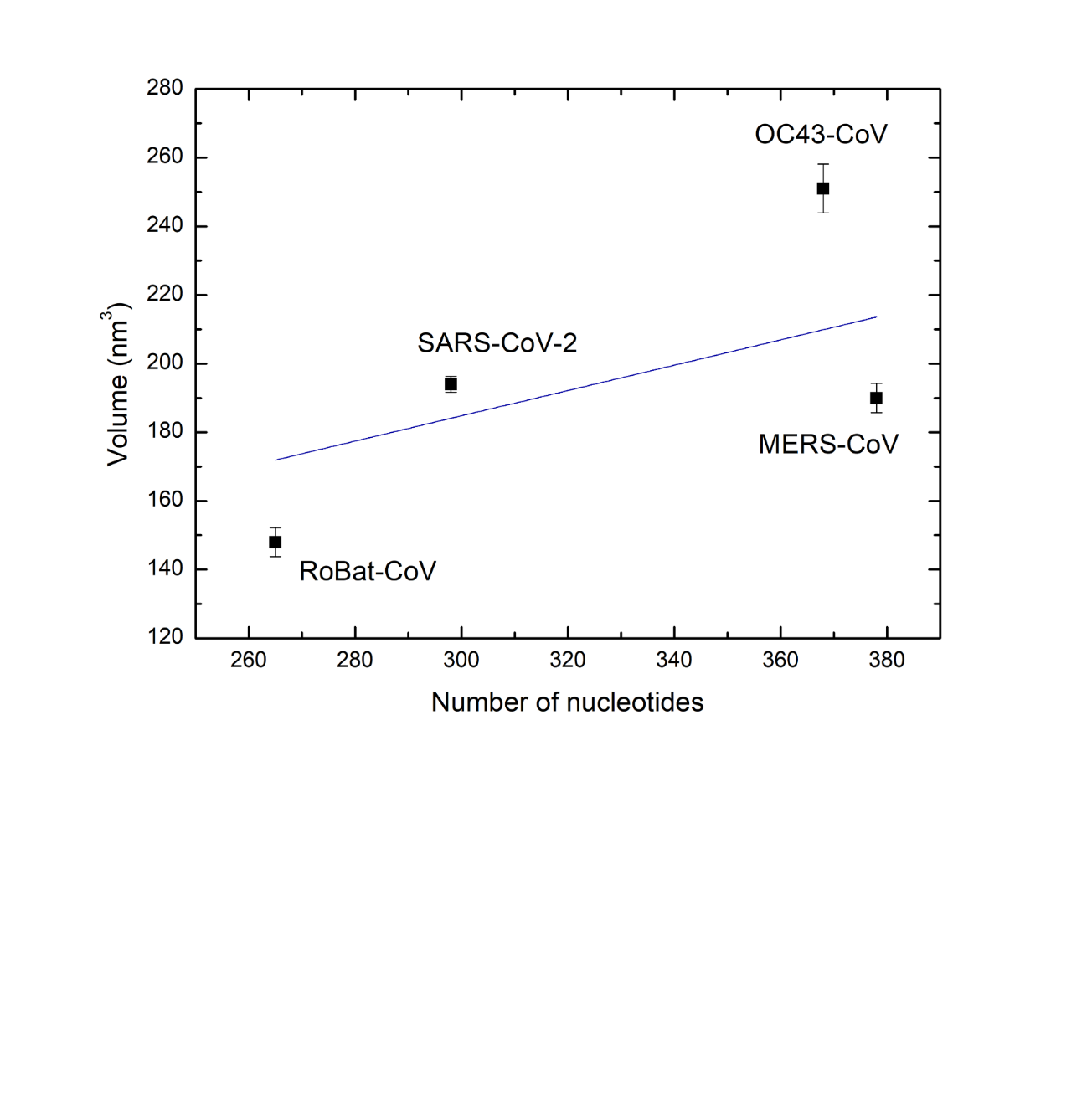


**Supplementary Figure 13: Mean volume of each sample as a function of the number of nucleotides.** Error bars represent the standard error of the mean.


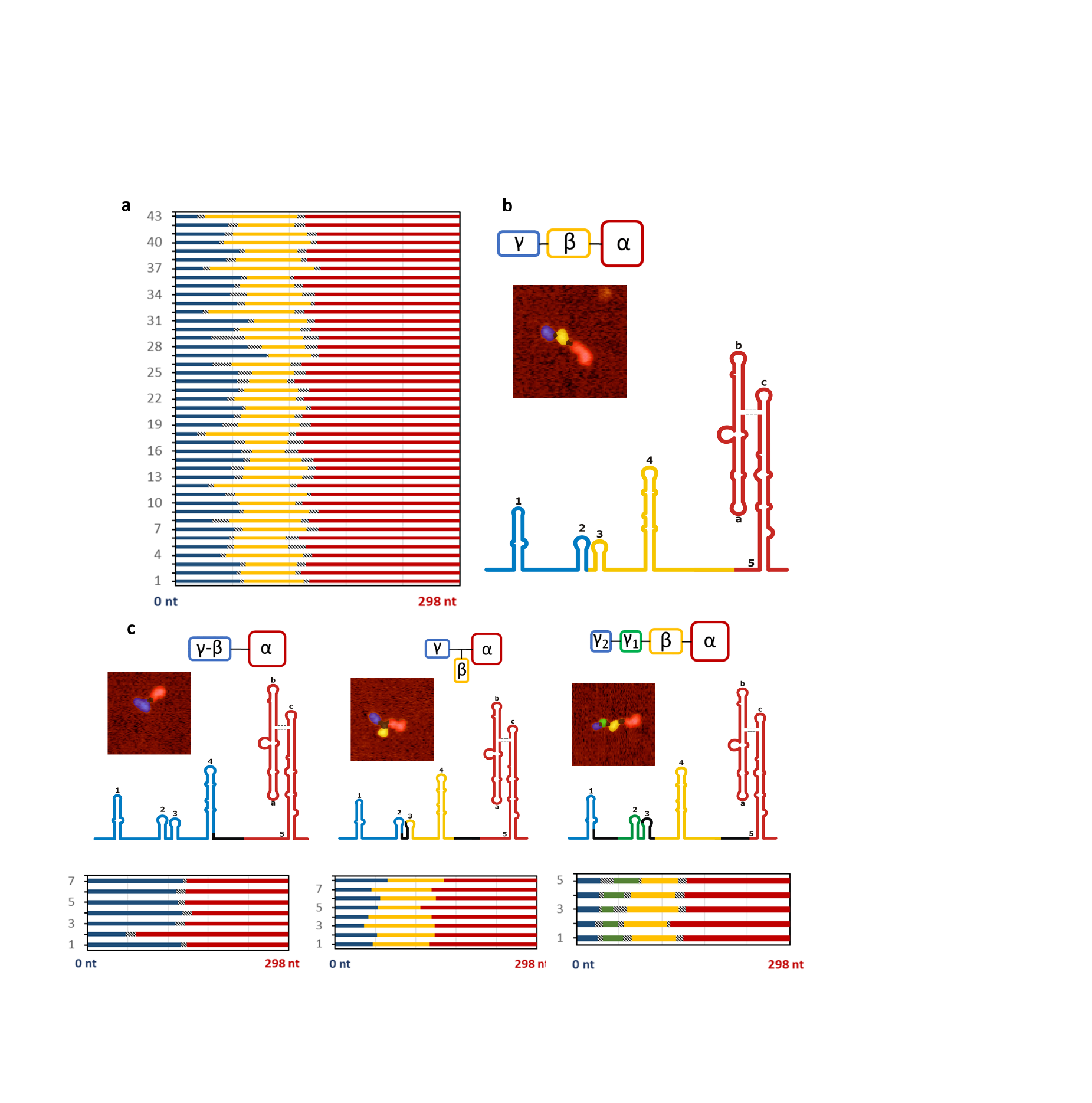


**Supplementary Figure 14:** **SARS-CoV-2 5′ proximal region in air AFM.** SARS-CoV-2 5′ proximal region shows homogeneous structures with 4 types of conformations. A) Volume maps of the type 2 conformation molecules b) nucleotide ranges obtained from averaging the volume maps of type 2 conformation superimposed to the secondary structure map c) Nucleotide ranges of the remaining 3 classes.


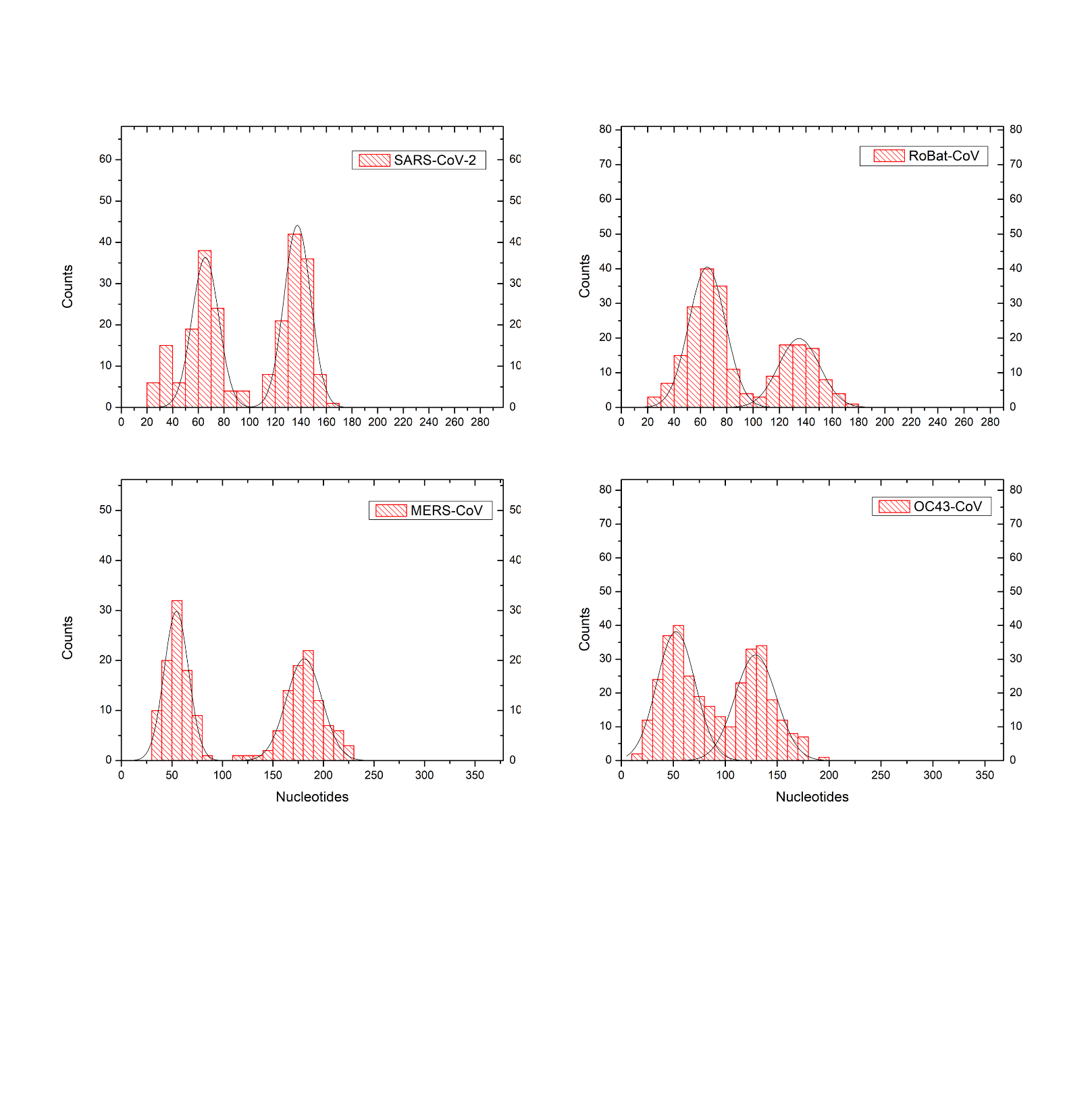


**Supplementary Figure 15: Distribution of boundaries of blob-like structures in sequences of the 5′-proximal regions of βCoV RNAs according to air AFM analyses.** All histograms present two-peaked distributions, which mean the three-blob molecules are the most common in all cases.


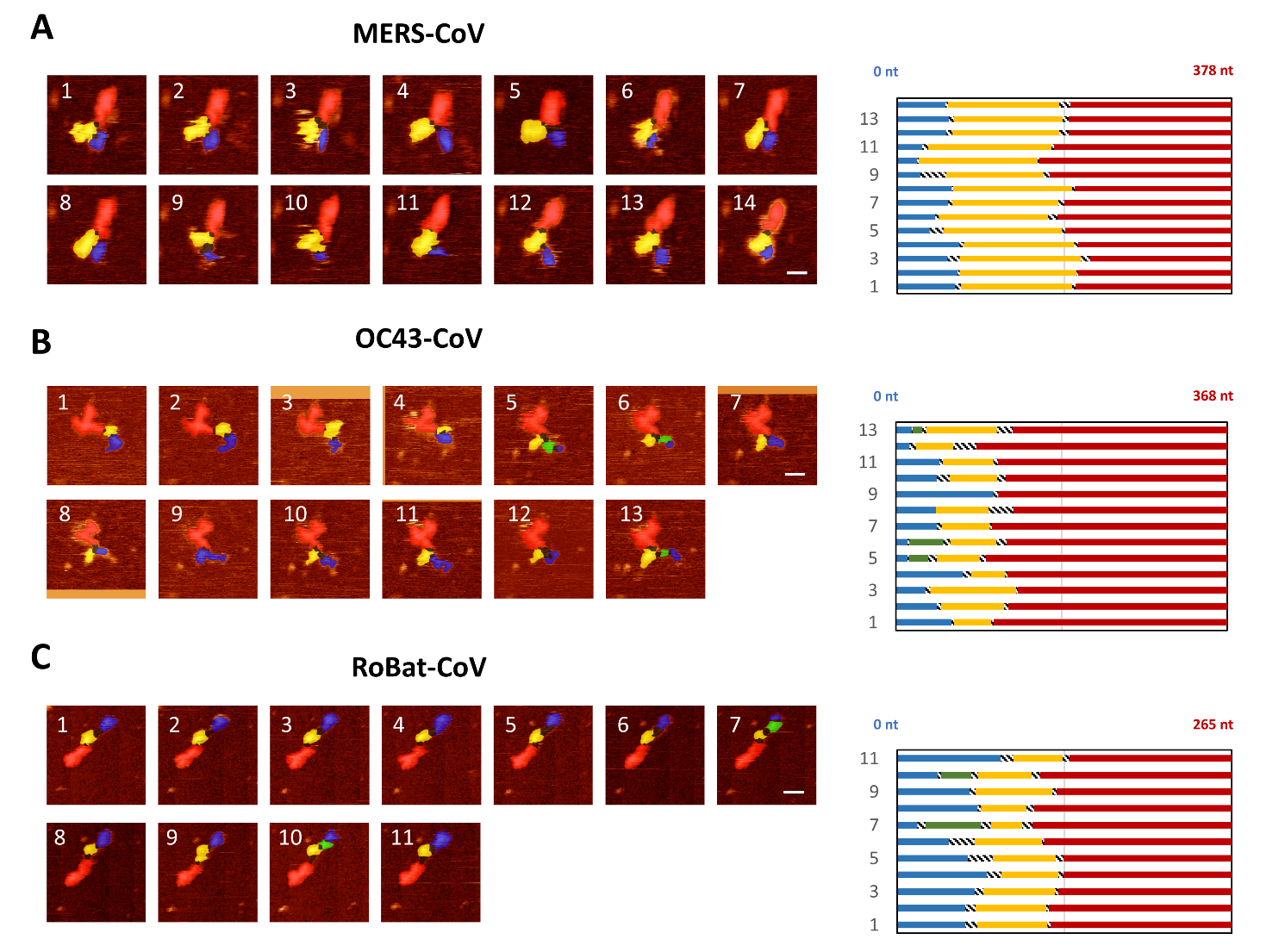


**Supplementary Figure 16: Liquid AFM imaging of MERS-CoV, OC43-CoV and RoBat-CoV.** A) Sequence of images of an individual MERS-CoV molecule taken in liquid environment (Supplementary Video 4, frame number indicated) and domain arrangement analysis. B) Sequence of images of an OC43-CoV molecule (Supplementary Video 5, frame number indicated) and domain arrangement analysis. C) Sequence of images of a RoBat-CoV molecule (Supplementary Video 6, frame number indicated) and domain arrangement analysis. The bar size is 20 nm, and the time interval between images is 1 minute.


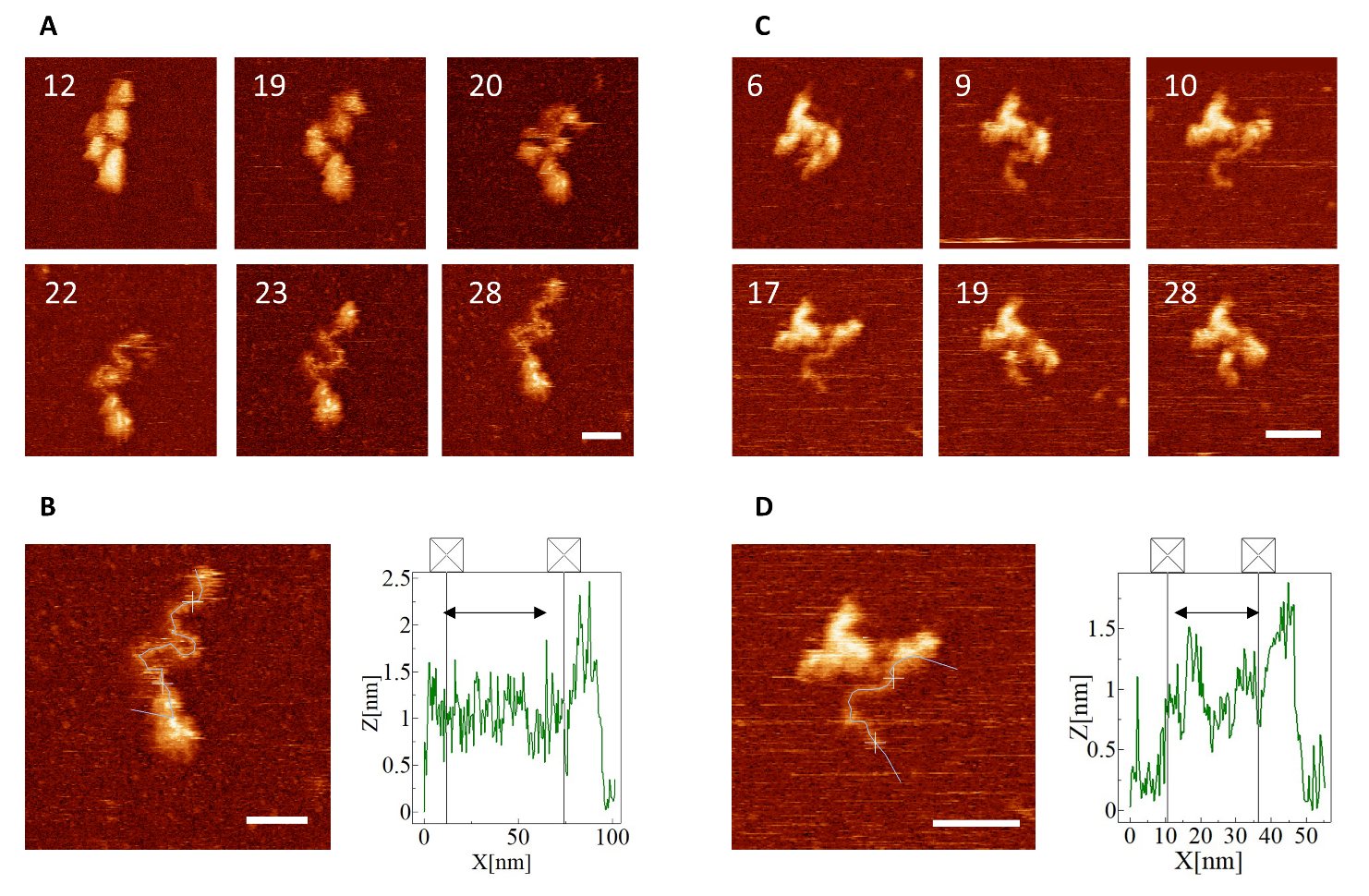


**Supplementary Figure 17: Cases of SARS-CoV-2 and OC43-CoV structure disassembly observed by AFM in solution.** A) Frames extracted from an AFM video taken in liquid environment, showcasing an individual SARS-CoV-2 molecule (Supplementary Video 2, with frame numbers indicated). Initially, 4 blob-like structures are observed, probably corresponding to SL1, SL2+SL3, SL4 and SL5. Notably, a sudden disassembly of the middle regions is observed. B) measured length of the single-stranded linked formed after the disassembly (62.5 nm). C) Frames extracted from an AFM video taken in liquid environment, showcasing an individual OC43 molecule (Supplementary Video 3, with frame numbers indicated). In this case, one of the terminal blob-like structures, likely corresponding to SL1, undergoes disassembly, resulting in a single stranded tail at the end of the molecule. D) The measured length of the single stranded region is 25.5 nm. The bar size is 20 nm, and the time interval between images is 1 minute.


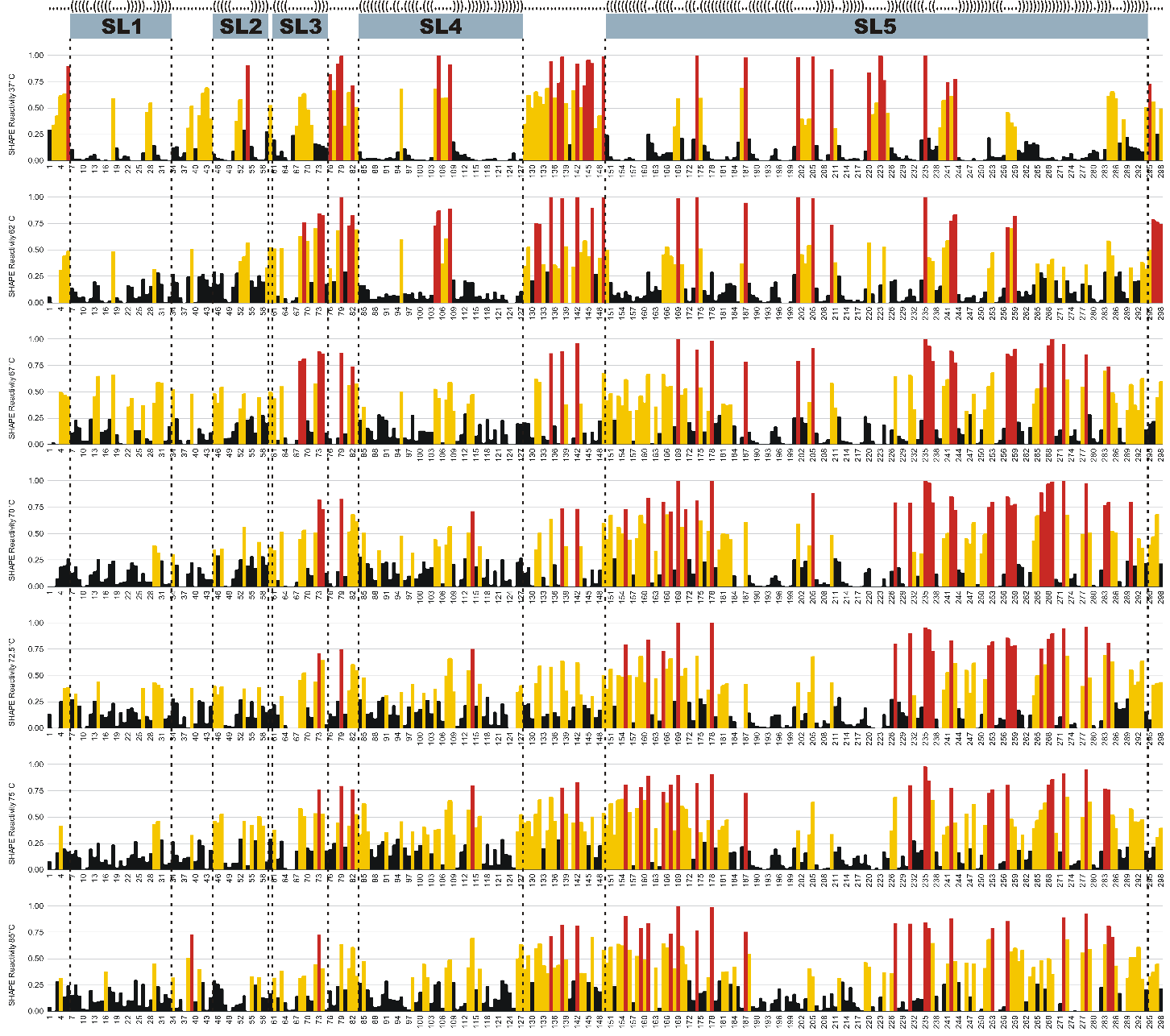
**Supplementary Figure 18:** **Reactivity plots for SARS-CoV-2 probing in temperature range of 62-85°C.** Reactivity plot for probing in 37°C is shown on top.

**Supplementary Table 1.** RNA sequences and DNA primers used in this study.

| **Name:** | **Sequence (5′ to 3′):** |
| --- | --- |
| **Sequences of the 5′-proximal regions of βCoV RNA genomes analyzed in this work** (*g*‘s added for the in vitro transcription) | |
| OC43-CoV | GAUUGUGAGCGAUUUGCGUGCGUGCAUCCCGCUUCACUGAUCUCUUGUUAGAUCUUUUUGUAAUCUAAACUUUAUAAAAACAUCCACUCCCUGUAAUCUAUGCUUGUGGGCGUAGAUUUUUCAUAGUGGUGUUUAUAUUCAUUUCUGCUGUUAACAGCUUUCAGCCAGGGACGUGUUGUAUCCUAGGCAGUGGCCCUCCCAUAGGUCACAAUGUCGAAGAUCAACAAAUACGGUCUCGAACUACACUGGGCUCCAGAAUUUCCAUGGAUGUUUGAGGACGCAGAGGAGAAGUUGGACAACCCUAGUAGUUCAGAGGUGGAUAUGAUUUGCUCCACCACUGCGCAAAAGCUGGAAACAGACGGAAUUUG |
| SARS-CoV-2 | *gg*AUUAAAGGUUUAUACCUUCCCAGGUAACAAACCAACCAACUUUCGAUCUCUUGUAGAUCUGUUCUCUAAACGAACUUUAAAAUCUGUGUGGCUGUCACUCGGCUGCAUGCUUAGUGCACUCACGCAGUAUAAUUAAUAACUAAUUACUGUCGUUGACAGGACACGAGUAACUCGUCUAUCUUCUGCAGGCUGCUUACGGUUUCGUCCGUGUUGCAGCCGAUCAUCAGCACAUCUAGGUUUCGUCCGGGUGUGACCGAAAGGUAAGAUGGAGAGCCUUGUCCCUGGUUUCAACGAGAAA |
| MERS-CoV | *g*GAUUUAAGUGAAUAGCUUGGCUAUCUCACUUCCCCUCGUUCUCUUGCAGAACUUUGAUUUUAACGAACUUAAAUAAAAGCCCUGUUGUUUAGCGUAUCGUUGCACUUGUCUGGUGGGAUUGUGGCAUUAAUUUGCCUGCUCAUCUAGGCAGUGGACAUAUGCUCAACACUGGGUAUAAUUCUAAUUGAAUACUAUUUUUCAGUUAGAGCGUCGUGUCUCUUGUACGUCUCGGUCACAAUACACGGUUUCGUCCGGUGCGUGGCAAUUCGGGGCACAUCAUGUCUUUCGUGGCUGGUGUGACCGCGCAAGGUGCGCGCGGUACGUAUCGAGCAGCGCUCAACUCUGAAAAACAUCAAGACCAUGUGUCUCUAACUGUGC |
| RoBat-CoV | *g*AUAAAAAGUAAUUGCGUGCGUGCAAUCAACUUUUCCCCCUCGAUUCGUCUUGUACGAUUCACUCUCUAACGAACUUAAAAUUGUGGCAUAUGUCACAGUGGGUUUCGUUCUGCUGUGCGUGUGCUACGCUUUGAUUUAAGUGAAGCUUUUGUGUCUUCAGCUUCCCUCUUCUCCUCGUGGGGUUCCGUUCCCGCGGUAGUGAGCCUGGUGUGGUUCCGCCCGCGCCAGUGAUGGAGGGUGCUUUAGGCACUAACAAGCUUAGAUC |
| **Sequences of the SL5 elements analyzed in this work** (*g*‘s added for the in vitro transcription) | |
| OC43-CoV | *gg*UUCUGCUGUUAACAGCUUUCAGCCAGGGACGUGUUGUAUCCUAGGCAGUGGCCCUCCCAUAGGUCACAAUGUCGAAGAUCAACAAAUACGGUCUCGAACUACACUGGGCUCCAGAAUUUCCAUGGAUGUUUGAGGACGCAGAGGAGAAGUUGGACAACCCUAGUAGUUCAGAGGUGGAUAUGAUUUGCUCCACCACUGCGCAAAAGCUGGAAACAGACGGAA |
| SARS-CoV-2 | *gg*UCGUUGACAGGACACGAGUAACUCGUCUAUCUUCUGCAGGCUGCUUACGGUUUCGUCCGUGUUGCAGCCGAUCAUCAGCACAUCUAGGUUUCGUCCGGGUGUGACCGAAAGGUAAGAUGGAGAGCCUUGUCCCUGGUUUCAACGA |
| MERS-CoV | *gg*UUUUUCAGUUAGAGCGUCGUGUCUCUUGUACGUCUCGGUCACAAUACACGGUUUCGUCCGGUGCGUGGCAAUUCGGGGCACAUCAUGUCUUUCGUGGCUGGUGUGACCGCGCAAGGUGCGCGCGGUACGUAUCGAGCAGCGCUCAACUCUGAAAAA |
| RoBat-CoV | *gg*AAGCUUUUGUGUCUUCAGCUUCCCUCUUCUCCUCGUGGGGUUCCGUUCCCGCGGUAGUGAGCCUGGUGUGGUUCCGCCCGCGCCAGUGAUGGAGGGUGCUUUAGGCACUAACAAGCUU |
| **Primers for assembly PCR:** | |
| SARS-CoV-2 -1F | ATTAAAGGTTTATACCTTCCCAGGTAACAAACCAACCAACTTTCGATCTCTTGTA |
| SARS-CoV-2 -2R | GCCACACAGATTTTAAAGTTCGTTTAGAGAACAGATCTACAAGAGATCGAAAGTTGGTTG |
| SARS-CoV-2 -3F | CGAACTTTAAAATCTGTGTGGCTGTCACTCGGCTGCATGCTTAGTGCACTCA |
| SARS-CoV-2 -4R | TGTCCTGTCAACGACAGTAATTAGTTATTAATTATACTGCGTGAGTGCACTAAGCATGCA |
| SARS-CoV-2 -5F | ACTGTCGTTGACAGGACACGAGTAACTCGTCTATCTTCTGCAGGCTGCTTACG |
| SARS-CoV-2 -6R | CGGCTGCAACACGGACGAAACCGTAAGCAGCCTGCAGAAGATA |
| SARS-CoV-2 -7F | CCGTGTTGCAGCCGATCATCAGCACATCTAGGTTTCGTCCGGGT |
| SARS-CoV-2 -8R | CCAGGGACAAGGCTCTCCATCTTACCTTTCGGTCACACCCGGACGAAACCTA |
| SARS-CoV-2 -9F | AGAGCCTTGTCCCTGGTTTCAACGAGAAATCGATCCGGTTCGCCGGATCCAAATCGGGC |
| SARS-CoV-2 -10R | GAACCGGACCGAAGCCCGATTTGGATCCGGCGA |
| MERS-CoV -1F | GATTTAAGTGAATAGCTTGGCTATCTCACTTCCCCTCGTTCTCTTGCAGAACTTTGA |
| MERS-CoV -2R | ACGCTAAACAACAGGGCTTTTATTTAAGTTCGTTAAAATCAAAGTTCTGCAAGAGAACG |
| MERS-CoV -3F | GCCCTGTTGTTTAGCGTATCGTTGCACTTGTCTGGTGGGATTGTGGCATTAATTTGCCTG |
| MERS-CoV -4R | ACCCAGTGTTGAGCATATGTCCACTGCCTAGATGAGCAGGCAAATTAATGCCACAATC |
| MERS-CoV -5F | ACATATGCTCAACACTGGGTATAATTCTAATTGAATACTATTTTTCAGTTAGAGCGTCGT |
| MERS-CoV -6R | GGACGAAACCGTGTATTGTGACCGAGACGTACAAGAGACACGACGCTCTAACTGAAAAAT |
| MERS-CoV -7F | ACAATACACGGTTTCGTCCGGTGCGTGGCAATTCGGGGCACATCATGTCTTTCGTGGCTG |
| MERS-CoV -8R | GCGCTGCTCGATACGTACCGCGCGCACCTTGCGCGGTCACACCAGCCACGAAAGACATGA |
| MERS-CoV -9F | CGTATCGAGCAGCGCTCAACTCTGAAAAACATCAAGACCATGTGTCTCTAACTGTGCTCG |
| MERS-CoV -10R | GAACCGGACCGAAGCCCGATTTGGATCCGGCGAACCGGATCGAGCACAGTTAGAGACACA |
| **Amplification of templates for RNA chemical probing:** | |
| 3pSC Rv | GAACCGGACCGAAGCCCGATTTGGATC |
| SARS-CoV-2 T75pSC | AATTTAATACGACTCACTATAGGGCCTTCGGGCCAAATTAAAGGTTTATACCTTCCCAGG |
| MERS-CoV  T75pSC | AATTTAATACGACTCACTATAGGGCCTTCGGGCCAAGATTTAAGTGAATAGCTTGGCTAT |
| RoBat-CoV _T7_SC | AATTTAATACGACTCACTATAGGGCCTTCGGGCCAAATAAAAAGTAATTGCGTGCG |
| OC43_SC_F | AATTTAATACGACTCACTATAGGGCCTTCGGGCCAAGATTGTGAGCGATTTGCG |
| OC43_3SC | CGAAGCCCGATTTGGATCCGGCGAACCGGATCGACAAATTCCGTCTGTTTCCAG |
| **Amplification of templates for native RNA production (full length and 4WJ):** | |
| SARS-CoV-2 T7GG | AATTTAATACGACTCACTATAGGATTAAAGGTTTATACCTTCCCAGG |
| SARS-CoV-2 Rv | TTTCTCGTTGAAACCAGGGACAAGGC |
| MERS-CoV T7G | AATTTAATACGACTCACTATAGGATTTAAGTGAATAGCTTGGCTATC |
| MERS-CoV Rv | GCACAGTTAGAGACACATGGTCTTG |
| RoBat-CoV _T7 | AATTTAATACGACTCACTATAGATAAAAAGTAATTGCGTGCG |
| RoBat-CoV _rev | GATCTAAGCTTGTTAGTGCC |
| OC43_F | AATTTAATACGACTCACTATAGATTGTGAGCGATTTGCG |
| OC43_R | CAAATTCCGTCTGTTTCCAGC |
| SARS-CoV-2_4WJ_Fv | TCGTTGACAGGACACGAG |
| SARS-CoV-2_4WJ_Rv | TCGTTGAAACCAGGGAC |
| SARS-CoV-2_4WJ_T7_Fv | AAATTAATACGACTCACTATAGGTCGTTGACAGGACACGAG |
| MERS-CoV _4WJ_Fv | TTTTTCAGTTAGAGCGTCG |
| MERS-CoV _4WJ_Rv | TTTTTCAGAGTTGAGCGCTG |
| MERS-CoV _4WJ_T7_Fv | AAATTAATACGACTCACTATAGGTTTTTCAGTTAGAGCGTCG |
| OC43_4WJ_Fv | TTCTGCTGTTAACAGCTTTC |
| OC43_4WJ_Rv | TTCCGTCTGTTTCCAGC |
| OC43_4WJ_T7_Fv | AAATTAATACGACTCACTATAGGTTCTGCTGTTAACAGCTTTC |
| RoBat-CoV _4WJ_F | AATTTAATACGACTCACTATAGGAAGCTTTTGTGTCTTCAGC |
| RoBat-CoV_4WJ_R | AAGCTTGTTAGTGCCTAAAGC |

**Supplementary Table 2.** Cryo-EM data collection, processing, and model refinement statistics.

|  | **OC43-CoV** | **SARS-CoV-2** | **MERS-CoV** | **RoBat-CoV** |
| --- | --- | --- | --- | --- |
| **Cryo-EM data collection and processing** | | | | |
| Microscope | Titan Krios | Titan Krios | Titan Krios | Titan Krios |
| Voltage (kV) | 300 | 300 | 300 | 300 |
| Detector | Gatan K3 | Gatan K3 | Gatan K3 | Gatan K3 |
| Electron exposure (e-/Å^2^) | 40 | 40 | 40 | 40 |
| Defocus range (μm) | 1.8 | -3.6 | -3.6 | 0.6 |
| Pixel size (Å) | 0.86 | 0.86 | 0.86 | 0.86 |
| Symmetry imposed | C1 | C1 | C1 | C1 |
| Micrographs (acquired/used) | 5,040/4,28 | 6,699/5,094 | 5,768/4,165 | 4,935/4,433 |
| Extracted particles | 408,666 | 343,981 | 376,173 | 340,645 |
| Particles after 2D classification | 389,967 | 220,419 | 262,104 | 242,284 |
| Particles going to 3D refinement | 65,145 | 29,234 | 46,032 | 44,519 |
| Map resolution at 0.143 FSC threshold (Å) | 7.07 | 6.5 | 5.89 | 6.61 |
| Local resolution range (Å) | 6.6-9.7 | 6.1-8.5 | 5.4 -7.2 | 6.2-8.2 |
| **Validation - Ensemble Range** | | | | |
| R.M.S.D. | 0.81 - 1.03 | 0.68 - 0.79 | 0.77 - 0.89 | 0.55 - 0.71 |
| MolProbity score | 3.04 - 3.15 | 3.03 - 3.11 | 3.08 - 3.15 | 2.99 - 3.15 |
| Clashscore | 4.3 - 6.7 | 1.0 - 5.8 | 3.1 - 6.4 | 3.4 - 6.1 |
| Poor rotamers (%) | 0 | 0 | 0 | 0 |
| **RNA geometry** | | | | |
| Bad bonds | 0 | 0 | 0 | 0 |
| Bad angles | 0 | 0 | 0 | 0 |
| Probably wrong sugar puckers | 0 - 1 | 0 - 1 | 0 - 1 | 0 - 1 |
| Q-score | 0.20 - 0.21 | 0.21 - 0.23 | 0.21 - 0.23 | 0.18 - 0.20 |

**Supplementary Table 3.** Nucleotide ranges extracted from AFM volume analysis. [mean(SD)]

Sequence ranges for class 1 molecules

| Sample | Big domain | Linker | End domain |
| --- | --- | --- | --- |
| OC43-CoV | 283 (37) | 17 (9) | 69 (35) |
| SARS-CoV-2 | 162 (29) | 12 (4) | 125 (31) |
| MERS-CoV | - | - | - |
| RoBat-CoV | 190 (23) | 13 (9) | 62 (21) |

Sequence ranges for class 2 molecules

| Sample | Big domain | Linker | middle domain | Linker | End domain |
| --- | --- | --- | --- | --- | --- |
| OC43-CoV | 232 (23) | 12 (7) | 59 (16) | 16 (7) | 48 (17) |
| SARS-CoV-2 | 159 (8) | 10 (3) | 64 (16) | 9 (6) | 57 (15) |
| MERS-CoV | 190 (16) | 14 (9) | 110 (16) | 12 (5) | 51 (13) |
| RoBat-CoV | 121 (16) | 16 (8) | 57 (13) | 13 (6) | 58 (14) |

Sequence ranges for class 3 molecules

| Sample | Big domain | Linker + middle domain | End domain |
| --- | --- | --- | --- |
| OC43-CoV | 201 (31) | 87 (25) | 81 (40) |
| SARS-CoV-2 | 153 (10) | 86 (12) | 59 (11) |
| MERS-CoV | 183 (15) | 141 (17) | 54 (14) |
| RoBat-CoV | 125 (12) | 79 (26) | 60 (14) |

Sequence ranges for class 4 molecules

| Sample | Big domain | Linker | middle domain | Linker | End domain 1 | linker | End domain 2 |
| --- | --- | --- | --- | --- | --- | --- | --- |
| OC43-CoV | 221 (17) | 13 (3) | 51 (8) | 17 (9) | 31 (7) | 7 (4) | 26 (9) |
| SARS-CoV-2 | 151 (9) | 10 (3) | 62 (7) | 11 (6) | 26 (7) | 10 (6) | 30 (3) |
| MERS-CoV | - | - | - | - | - | - | - |
| RoBat-CoV | - | - | - | - | - | - | - |

Sequence ranges for molecules of all classes

| Sample | 5′ domain | Middle | 3′ domain |
| --- | --- | --- | --- |
| OC43-CoV | 0 to 53 (18) | 54 (18) to 129 (20) | 130 (20) to 368 |
| SARS-CoV-2 | 0 to 66 (10) | 67 (10) to 137 (10) | 138 (10) to 298 |
| MERS-CoV | 0 to 54 (12) | 55 (12) to 181 (18) | 182 (18) to 378 |
| RoBat-CoV | 0 to 65 (14) | 66 (14) to 135 (16) | 136 (16) to 265 |

**Supplementary Table 4.** Melting temperatures of all four βCoVs 5′-proximal regions and junctions.

| Tm |  | | Mean (°C) | Standard Deviation |
| --- | --- | --- | --- | --- |
|  | OC43-CoV | SL5 element | 61.64 | 0.06 |
|  |  | 5′-Proximal Region | 62.21 | 0.04 |
|  | SARS-CoV-2 | SL5 element | 62.06 | 0.13 |
|  |  | 5′-Proximal Region | 62.14 | 0.02 |
|  | MERS-CoV | SL5 element | 60.91 | 0.02 |
|  |  | 5′-Proximal Region | 60.40 | 0.05 |
|  | RoBat-CoV | SL5 element | 63.60 | 0.12 |
|  |  | 5′-Proximal Region | 66.24 | 0.04 |

**Supplementary Table 5.** RMSD values of SL5 core junction superpositions.

|  | Percentage of the shortest chain residues aligned | | | |
| --- | --- | --- | --- | --- |
|  | OC43-CoV | SARS-CoV-2 | MERS-CoV | RoBat-CoV |
| OC43-CoV | - | 95.00% | 91.00% | 81.00% |
| SARS-CoV-2 | - | - | 100.00% | 78.00% |
| MERS-CoV | - | - | - | 78.00% |
| RoBat-CoV | - | - | - | - |
|  |  |  |  |  |
|  | RMSD | | | |
|  | OC43-CoV | SARS-CoV-2 | MERS-CoV | RoBat-CoV |
| OC43-CoV | - | 2.6 | 2.99 | 3.69 |
| SARS-CoV-2 | - | - | 2.36 | 2.95 |
| MERS-CoV | - | - | - | 3.45 |
| RoBat-CoV | - | - | - | - |

**SUPPLEMENTARY REFERENCES**

1. Lee,C.W., Li,L. and Giedroc,D.P. (2011) The solution structure of coronaviral stem-loop 2 (SL2) reveals a canonical CUYG tetraloop fold. *FEBS Lett*, **585**, 1049–1053.

2. Vögele,J., Hymon,D., Martins,J., Ferner,J., Jonker,H.R.A., Hargrove,A.E., Weigand,J.E., Wacker,A., Schwalbe,H., Wöhnert,J., *et al.* (2023) High-resolution structure of stem-loop 4 from the 5′-UTR of SARS-CoV-2 solved by solution state NMR. *Nucleic Acids Res*, **51**, 11318–11331.

3. Manfredonia,I., Nithin,C., Ponce-Salvatierra,A., Ghosh,P., Wirecki,T.K., Marinus,T., Ogando,N.S., Snijder,E.J., van Hemert,M.J., Bujnicki,J.M., *et al.* (2020) Genome-wide mapping of SARS-CoV-2 RNA structures identifies therapeutically-relevant elements. *Nucleic Acids Res*, **48**, 12436–12452.

4. Wacker,A., Weigand,J.E., Akabayov,S.R., Altincekic,N., Bains,J.K., Banijamali,E., Binas,O., Castillo-Martinez,J., Cetiner,E., Ceylan,B., *et al.* (2020) Secondary structure determination of conserved SARS-CoV-2 RNA elements by NMR spectroscopy. *Nucleic Acids Res*, **48**, 12415–12435.

5. Huston,N.C., Wan,H., Strine,M.S., de Cesaris Araujo Tavares,R., Wilen,C.B. and Pyle,A.M. (2021) Comprehensive in vivo secondary structure of the SARS-CoV-2 genome reveals novel regulatory motifs and mechanisms. *Mol Cell*, **81**, 584-598.e5.

6. Richter,C., Hohmann,K.F., Toews,S., Mathieu,D., Altincekic,N., Bains,J.K., Binas,O., Ceylan,B., Duchardt-Ferner,E., Ferner,J., *et al.* (2021) 1H, 13C and 15N assignment of stem-loop SL1 from the 5’-UTR of SARS-CoV-2. *Biomol NMR Assign*, **15**, 467–474.

7. Rother,M., Rother,K., Puton,T. and Bujnicki,J.M. (2011) ModeRNA: a tool for comparative modeling of RNA 3D structure. *Nucleic Acids Res*, **39**, 4007–4022.
