# Supplementary material for: Conserved structures and dynamics in 5′-proximal regions of Betacoronavirus RNA genomes
Source: Nucleic Acids Res. 2024 Mar 1;52(6):3419–32. doi: 10.1093/nar/gkae144 (PMC11014237; doi:10.1093/nar/gkae144)
Supplement: gkae144_Supplemental_Files [file gkae144_supplemental_files.zip › NAR-03013-H-2023_AFM-VIDEOS-legends.docx]

**AFM SUPPLEMENTARY VIDEOS LEGENDS**

**Supplementary Video 1: SARS-CoV-2 RNA in solution.** Consecutive AFM images of a single RNA molecule of a 5′-proximal region of the SARS-CoV-2 genome taken in solution (17 frames). Dynamic arrangement of structural elements and flexible linkers can be observed. Image size: 60x60 nm. Image rate: ~1 image per minute.

**Supplementary Video 2: SARS-CoV-2 RNA in solution, an unfolding event of a structured element.** Consecutive AFM images of a single RNA molecule of a 5′-proximal region of the SARS-CoV-2 genome taken in solution (26 frames). The unfolding of one structured element can be observed. Image size: 80x80 nm. Image rate: ~1 image per minute.

**Supplementary Video 3: OC43-CoV RNA in solution, an unfolding event of a structured element.** Consecutive AFM images of a single RNA molecule of a 5′-proximal region of the OC43-CoV genome taken in solution (27 frames). The unfolding of one structured element can be observed. Image size: 70x70 nm. Image rate: ~1 image per minute.

**Supplementary Video 4: MERS-CoV RNA in solution.** Consecutive AFM images of a single RNA molecule of a 5′-proximal region of the MERS-CoV genome taken in solution (14 frames). Dynamic arrangement of structural elements and flexible linkers can be observed. Image size: 60x60 nm. Image rate: ~1 image per minute.

**Supplementary Video 5: OC43-CoV RNA in solution.** Consecutive AFM images of a single RNA molecule of a 5′-proximal region of the OC43-CoV genome taken in solution (13 frames). Dynamic arrangement of structural elements and flexible linkers can be observed. Image size: 60x60 nm. Image rate: ~1 image per minute.

**Supplementary Video 6: RoBat-CoV RNA in solution.** Consecutive AFM images of a single RNA molecule of a 5′-proximal region of the RoBat-CoV genome taken in solution (11 frames). Dynamic arrangement of structural elements and flexible linkers can be observed. Image size: 60x60 nm. Image rate: ~1 image per minute.
